# Transcriptional analysis of *Ceratopteris richardii* young sporophyte reveals conservation of stem cell factors in the root apical meristem

**DOI:** 10.3389/fpls.2022.924660

**Published:** 2022-08-11

**Authors:** Alejandro Aragón-Raygoza, Luis Herrera-Estrella, Alfredo Cruz-Ramírez

**Affiliations:** ^1^Molecular and Developmental Complexity Group, Unidad De Genómica Avanzada, Laboratorio Nacional De Genómica Para la Biodiversidad, Cinvestav Unidad Irapuato, Irapuato, Guanajuato, Mexico; ^2^Metabolic Engineering Group, Unidad De Genómica Avanzada, Laboratorio Nacional De Genómica Para la Biodiversidad, Cinvestav Unidad Irapuato, Irapuato, Guanajuato, Mexico; ^3^Department of Plant and Soil Science, Institute of Genomics for Crop Abiotic Stress Tolerance, Texas Tech University, Lubbock, TX, United States

**Keywords:** *Ceratopteris richardii*, root development, gene expression, gene evolution, transcriptome, ground tissue, root cap

## Abstract

Gene expression in roots has been assessed in different plant species in studies ranging from complete organs to specific cell layers, and more recently at the single cell level. While certain genes or functional categories are expressed in the root of all or most plant species, lineage-specific genes have also been discovered. An increasing amount of transcriptomic data is available for angiosperms, while a limited amount of data is available for ferns, and few studies have focused on fern roots. Here, we present a *de novo* transcriptome assembly from three different parts of the *Ceratopteris richardii* young sporophyte. Differential gene expression analysis of the root tip transcriptional program showed an enrichment of functional categories related to histogenesis and cell division, indicating an active apical meristem. Analysis of a diverse set of orthologous genes revealed conserved expression in the root meristem, suggesting a preserved role for different developmental roles in this tissue, including stem cell maintenance. The reconstruction of evolutionary trajectories for ground tissue specification genes suggests a high degree of conservation in vascular plants, but not for genes involved in root cap development, showing that certain genes are absent in Ceratopteris or have intricate evolutionary paths difficult to track. Overall, our results suggest different processes of conservation and divergence of genes involved in root development.

## Introduction

The appearance of roots during plant evolution was a cornerstone event that shaped current ecosystems. The importance of this organ relies on its diverse and important functions for plant survival, such as providing mechanical anchorage, nutrient and water uptake, and interaction with soil microorganisms, among others. As a complex organ, the root represents a major trait that played a crucial role in the adaptation of plants to dynamically changing environments and led vascular plants to become the dominant vegetation in our planet (Friedman et al., 2004; Pires and Dolan, 2012).

Root morphology has been comprehensively analyzed in different plant lineages (Heimsch and Seago, 2008; Seago and Fernando, 2013). Previous studies showed that the roots of all vascular plants are composed of similar structures and layers: a stem cell niche, vascular tissue (pericycle, phloem and xylem), ground tissue (cortex and endodermis), an epidermis, and a root cap (Bennett and Scheres, 2010; Kumpf and Nowack, 2015). However, root developmental programs have only been characterized in detail in *Arabidopsis thaliana*. The Arabidopsis root has been studied from an initial morphological description and characterization of genes involved in root patterning, to complex gene expression analysis that unraveled the transcriptional landscapes of individual cell layers (Dolan et al., 1993; Scheres et al., 1994; Birnbaum et al., 2003; Brady et al., 2007; Denyer et al., 2019; Jean-Baptiste et al., 2019; Ryu et al., 2019; Shulse et al., 2019; Zhang et al., 2019; Wendrich et al., 2020). However, there is still a considerable gap between the knowledge on Arabidopsis root development, that on the evolution of the root transcriptional program and the molecular regulation of root growth in other lineages, such as lycophytes, ferns, and gymnosperms (Augstein and Carlsbecker, 2018; Motte et al., 2020).

Few studies have focused on analyzing how genes and their expression patterns are conserved among the roots of different plant lineages. Root transcriptomics, under the premise of plant diversity, would allow understanding the transcriptional landscape of this organ in a phylogenetic context (Huang and Schiefelbein, 2015; Ferrari et al., 2020; Kajala et al., 2021). Comparative transcriptomic analyses revealed that the expression of the Arabidopsis root core genes is generally conserved in the different root developmental zones of diverse plant species, including angiosperms and lycophytes (Huang and Schiefelbein, 2015). Also, most genes shared among root transcriptomes, predates the appearance of this organ in vascular plants, suggesting that little genetic novelty was necessary during the evolution of roots (Ferrari et al., 2020).

Ferns were the last lineage of vascular plants to be considered for genome sequencing due to their high chromosome number and large genome size (Barker and Wolf, 2010; Wolf et al., 2015; Sessa and Der, 2016). To date, the genome of only four different fern species is available, *Alsophila spinulosa*, *Azolla filiculoides* (Azolla), *Ceratopteris richardii* (Ceratopteris), and *Salvinia cucullata* (Li et al., 2018; Marchant et al., 2019; Huang et al., 2022). While genomes from other ferns species will be available in the near future (Kinosian and Wolf, 2022).

*Ceratopteris richardii* is the only fern species from of the order Polypodiales, the major fern lineage, to have a complete sequenced genome and where genetic transformation has been established (Plackett et al., 2014; Bui et al., 2015; Marchant, 2019; Marchant et al., 2019). Its development from spores to whole fertile sporophytes has been recently characterized, including the root system structure and development during different stages of the sporophyte (Hou and Hill, 2002, 2004; Conway and Di Stilio, 2019; Aragón-Raygoza et al., 2020). This opens the possibility to use Ceratopteris as fern model organism for root developmental analysis using novel genetic and molecular tools. Several gene expression analyses have been performed in Ceratopteris to understand spore germination, gametophyte development, sexual determination, and leaf development (Salmi et al., 2005; Cao et al., 2010; Bushart et al., 2013; Bui et al., 2017; Atallah et al., 2018; Geng et al., 2021). In the case of root development, some Ceratopteris transcriptomes have included this organ in their datasets while not exploring the full potential of the generated data or lacking replicates to perform differential expression analyses (Yu et al., 2020; Geng et al., 2021).

Here, we report a robust *de novo* transcriptome assembly from three different parts of *C. richardii* young sporophyte. While we focused on the root tip, the differential expression analysis showed that the root meristem is enriched in gene ontology categories related to organ development and the mitotic cell cycle. Also, we detected the expression of certain gene families specifically in the root tip compared to the other tissues. We also report that the gene circuit implicated in the cortex-endodermis specification of Arabidopsis is also present in Ceratopteris while all the members are expressed in the root tip. In summary, our transcriptional approach represents a valuable resource to explore the conservation of genes that are key regulators during developmental processes in the root but also in the shoot.

## Materials and methods

### Plant material and growth

*Ceratopteris richardii* cultivar Hn-n spores were cultured as described in Aragón-Raygoza et al. (2020). Young sporophytes from 15 days after fertilization (daf) were sub-culture in CFM (C-Fern Medium) plus 2% sucrose, 0.5 g/L MES, 200 mg/L CaCl_2_, 200 mg/L MgSO_4_·7H_2_O, pH 6.0, and 0.4% Gellan Gum. Plants were grown vertically at 25°C with a photoperiod of 16/8 h light/dark until the third stem-borne root emerged. Ceratopteris sporophytes were dissected into three different fractions: leaves and stem (shoot, SH), roots without the tip (DT), and root tip (RT). From the shoots, we were careful to remove any possible stem-borne root tissue; this sample only comprises young and rounded leaves with the main stem. To dissect the root tip, we cut below the visible strands of vascular tissue. Tissues were frozen in liquid nitrogen right after the plant dissection. For RNA isolation we used the shoots from 10 plants, 100 roots without their tip, and 250 root tips. The samples were stored at −80°C until processed.

### RNA isolation and quality assessment

Plant organs were grounded in the presence of liquid nitrogen to a fine powder. Samples were transferred to PureLink® Plant RNA Reagent and mixed by inversion until no visible clumps of tissue were observed. We followed the recommended method from the manufacturer, except for the overnight incubation of the aqueous phase with isopropyl alcohol at 4°C. Samples were resuspended in 50 μl of DEPC-treated water. RNA quantification was carried out using a NanoDrop 2000 Spectrophotometer. RNA quality was assessed by electrophoresis with a 1% agarose gel dissolved in 1X TAE buffer (DEPC-treated). After, the samples were treated with DNAse I to eliminate DNA contamination in the following procedures.

### Library construction and sequencing

Three independent biological replicates per plant fragment were used to construct the libraries and then sequenced. The RNA integrity of each sample was evaluated with a BioAnalyzer 2100 device. Samples were processed to generate sequencing libraries using the TruSeq RNA Prep Kit V2. The libraries were sequenced using the Illumina NextSeq 500 system to generate 150 bp paired-end reads. We obtained an average of 32 million paired end reads per sample. All these procedures were carried out at the Laboratorio de Servicios Genómicos (LABSERGEN) from the Advanced Genomics Unit (LANGEBIO).

### Data assessment and processing

We assessed the quality of the raw sequencing data with FastQC v.0.11.2 (Andrews, 2010). Sequencing data were then processed using Trimmomatic v0.32 to discard unpaired reads, reads with a size <60 bp, reads with a quality <20, and to remove the adapters (Bolger et al., 2014).

### *De novo* transcriptome assembly

Sequencing data from all libraries were used to generate a complete *de novo* transcriptome from *C. richardii* young sporophyte. The transcriptome was assembled with Trinity v2.4.0 in the default setting without reads normalization (Grabherr et al., 2011; Haas et al., 2013). After the transcriptome was obtained, we analyzed the basic stats using Perl scripts from the Trinity package and quantified the read usage with Bowtie2 v2.3.4.2 (Langmead and Salzberg, 2012). We assessed the transcriptome completeness by using BUSCO v3 databases for plants: Embryophyta *odb9*; Embryophyta *odb10*; and Viridiplantae *odb10* (Seppey et al., 2019). Also, we integrated two different approaches to cluster the transcriptome sequences: (1) sequence similarity with CD-HIT v4.6with a sequence identity of 95%; (2) read usage with Compacta v.1.01 with default settings (Fu et al., 2012; Razo-Mendivil et al., 2020). The output was established as the final version of the transcriptome.

### Transcriptome annotation

First, we determined the different coding sequences of the transcriptome using Transdecoder v.5.3.0.^1^ Then, we searched sequence similarity using NCBI BLAST+ v.2.1.31 against different databases: Viridiplantae reviewed sequences from SwissProt; *A. thaliana* reference proteome from Uniprot; *A. thaliana* proteome Araport11 from Phytozome; *A. filiculoides* and *S. cucullata* from FernBase (Camacho et al., 2009). We also used SignalP v4.1, tmhmm v.2.0, RNAmmer v1.2, and tRNAscan-SE v2.0 (Krogh et al., 2001; Lagesen et al., 2007; Nielsen, 2010; Chan and Lowe, 2019). All the generated outputs were put together with Trinotate v.3.2.1 in a single file for later purposes (Bryant et al., 2017).

### Differential gene expression analysis

The transcript abundance from each sample was quantified with kallisto v.0.44.0 from processed reads against the final version of Ceratopteris transcriptome (Bray et al., 2016). We exported each dataset with the R package tximport to facilitate data usage (Soneson et al., 2016). We took the raw read counts for each sample for data processing and normalization with the R package edgeR v.3.32.1 (Robinson et al., 2009; McCarthy et al., 2012). We removed genes with low read counts across all samples by using filterByExpr() function and normalized read counts according to the effective library size for each sample. Then, we performed the gene expression analysis with the R package limma v.3.46.0 while using the voom function along with other functions to calculate differential gene expression between the different tissues and contrasts, such as lmfit, contrasts.fit, and ebayes (Law et al., 2014, 2018; Ritchie et al., 2015). Plots were generated using the R packages ggplot2, pheatmap, UpSetR, and viridis (Conway et al., 2017).

### Gene ontology enrichment analysis

The annotation data from the transcriptome were imported with the R package trinotateR, since it included gene ontology (GO) annotations for each gene. Genes were considered to be upregulated in a specific tissue if they had a log2 fold change (FC) value ≥2 and a FDR adjusted *p* value (adj.P.Val) ≤ 0.05. On the contrary, genes with a FC value of ≤2 were considered to be downregulated. Only genes that fulfill the filtering criteria were included to analyze enriched GO terms into the R package topGO. Plots were generated using the R packages ggplot2 and viridis.

### Transcription factor family analysis

We selected transcripts that had a PFAM domain associated with transcription factors and that matched with Arabidopsis transcript factor (TF). Then, TF-coding genes were considered to be differentially expressed in a specific tissue if they FC value ≤ or ≥2 and an adj.P.Val ≤ 0.05. The transcripts were clustered by TF family and expression type (upregulated or downregulated). The mean FC value was calculated according to the expression type in each TF family. Plots were generated using the R packages ggplot2 and colorspace (Wickham, 2016).

### Comparative analysis with other *Ceratopteris* dataset

Another dataset from different Ceratopteris sporophyte parts was generated in recent study (Yu et al., 2020). We compared these samples with the ones generated in this work. The samples from that report were assessed, processed, and analyzed in a similar manner as we did with our own samples. Since both datasets were collected and processed in different laboratories, we assessed this issue by including a correction of the batch effect between datasets with the removeBatchEffect function (Kajala et al., 2021). This correction was only used when we analyze the similarities and differences between samples with a multidimensional scaling analysis (MDS). Gene ontology analysis was performed similarly as mentioned above. Plots were generated using the R packages ggplot2, pheatmap, UpSetR, and viridis (Conway et al., 2017).

### Gene phylogenetic reconstruction

We assessed the assignment of orthologs using OrthoFinder v.2.4.0 along with NCBI BLAST+ v.2.1.31 and MAFFT v.7305 (Camacho et al., 2009; Emms and Kelly, 2018; Katoh et al., 2018). We looked for the orthogroup ID which contained the Arabidopsis gene of interest. After that, we collected the protein sequences of selected species. We also search for aminoacidic sequences of other ferns using the OneKP database to enrich this clade (One Thousand Plant Transcriptomes Initiative, 2019). Sequences were aligned using MAFFT online server^2^ and selecting a specific iterative refinement method (E-INS-i or L-INS-i) according to the protein domain composition (Katoh et al., 2018). Manual editing was performed for each alignment using the Jalview software. The aminoacidic substitution model was obtained from ModelTest-NG (Darriba et al., 2020). Phylogenetic reconstruction was assessed with maximum likelihood in the IQ-Tree software along with ultrafast bootstrapping of 5,000 repetitions in CIPRES Science Gateway v3.3 (Miller et al., 2010; Hoang et al., 2017; Minh et al., 2020). Tree edition was carried out in iTOL v4 (Letunic and Bork, 2019).

### Gene expression between tissues

We selected the Ceratopteris orthologs for well-known Arabidopsis root genes from the phylogenetic reconstructions. Then, we searched for their fold change expression in each plant fragment of the transcriptome analysis. Transcripts were filtered according to an adj.P.Val ≤ 0.05. Plots were generated using the R packages ggplot2 and colorspace (Wickham, 2016).

## Results

### A *Ceratopteris richardii* transcriptome from the young sporophyte

We generated a *de novo* transcriptome assembly from three different parts of 45-daf sporelings with active roots: the root tip (RT), roots without their tips (differentiated root, DR), and the shoot (leaves and stem, SH). Our transcriptome was generated by sequencing total RNA libraries from three independent biological replicates per *Ceratopteris* segment. ~280 M paired-end reads were sequenced and used for the assembly of this *Ceratopteris* transcriptome (Table 1). We obtained a transcriptome with >300 K transcripts with an average length of 970 bp, considering contigs with an assembled length of ≥200 bp (Table 1). Since this number of transcripts may represent different gene isoforms, we decided to use two different clustering methods to compact the transcriptome size. After these steps, we achieved a transcriptome of >150 K contigs, including >40 K coding sequences (CDS).

**Table 1:** Basic information of the *de novo Ceratopteris richardii* transcriptome from young sporophyte segments.

*Ceratopteris richardii* Hn-n	
**Whole RNA (Bulk) Transcriptome**	
**Plant segments:**	
Shoot (leaves & stem)	
Root (without tip)	
Root tip	
**Transcriptome Assembly:**	
Trinity v.2.4.0	
kmer size = 25	
**Basic Stats:**	
Total Trinity transcripts	308,816
Total Trinity “genes”	183,202
Total CDHIT transcripts (>95% coverage >95% similarity)	238,142
Total Compacta transcripts	181,185
**Transcriptome v1.0**	
Total transcripts	154,546
Total CDS	38,427

During clusterization, we assessed if the reduction of contigs affected the transcriptome composition. Based on searches in the BUSCO Plants databases, we only observed a reduction in the number of duplicated genes (from ~30–45 to <5%) and an increase in single-copy genes (from ~35–50 to 55–85%; Supplementary Figure 1A). This could indicate that the transcriptome was highly redundant and that mRNA isoforms from the same gene or artifacts from the *de novo* assembly were present. Also, in comparison with previous Ceratopteris transcriptomes, our transcriptome had a larger number of coding transcripts than those previously reported (Supplementary Figure 1B), which could imply technical factors (deeper and more complete profiling due to the technical replicates and fragmentation of actual genes) or developmental implications (high diversity of transcripts and/or isoforms per organ, tissue diversity in each fragment, and organ differentiation processes).

**Figure 1:**
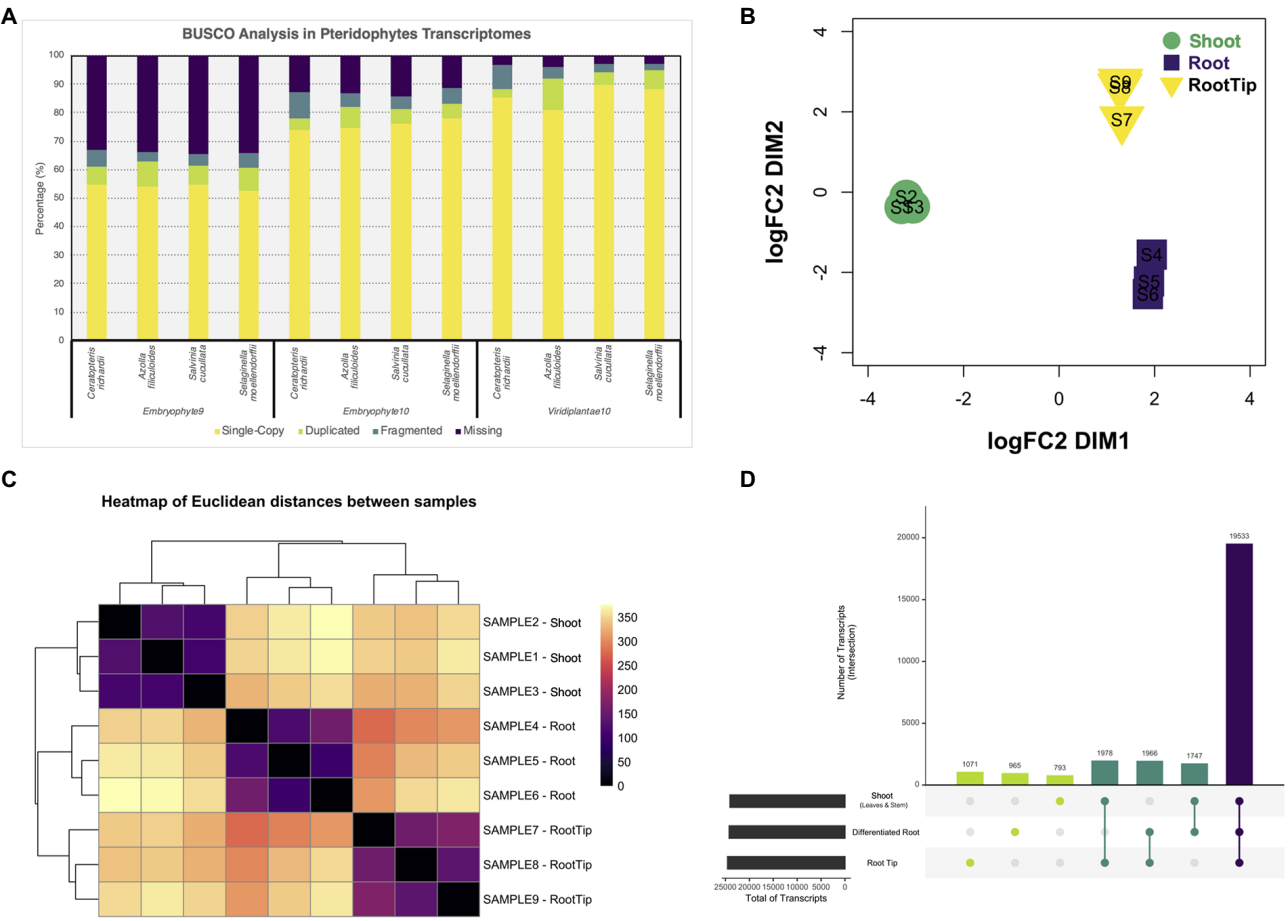
Differential gene expression analysis from tissues of *Ceratopteris richardii* young sporophyte. **(A)** Comparison of the gene content between pteridophytes transcriptomes using different BUSCO databases. **(B)** Multidimensional scaling analysis to examine how the different samples and segments are grouped between one another. **(C)** Heatmap showing the grouping between samples and segments. **(D)** Plot showing the unique and shared transcripts between plant parts.

To assess the quality of our transcriptome, we analyzed its completeness with three different BUSCO Plants databases and compared it with the predicted transcriptomes of other pteridophytes and lycophytes (Figure 1A). Also, we contrasted the different versions of our transcriptome with both available versions of *Ceratopteris* genome (Supplementary Figure 1C; Marchant et al., 2019, version 2 available in Phytozome v13). While our data showed improved gene completeness than the first genome version, the transcriptome predicted from the newest genome assembly had less fragmented BUSCO hits and showed a higher level of duplicated genes (Marchant et al., 2019). In all the cases, we observed similar values of gene content, which suggests that our transcriptome is well-assembled, contain the majority of well-conserved orthologs, and could be used for downstream analysis.

### Differential gene expression analysis of the plant segments in *Ceratopteris* sporophyte

After generating a high-confidence global transcriptome, we analyzed gene expression at the organ level. We performed a multidimensional scaling analysis to examine the grouping between samples and plant segments (Figure 1B). The samples were arranged together according to the plant section without any outlier. We also observed that root tip and differentiated root grouped closer to each other than to the shoot (Figure 1C). Both RT and DR come from the root, and this result demonstrates that they are closer because they are part of the same organ, while they separate base on different developmental stages and/or maturation status.

Then, we performed differential gene expression analysis to determine genes that are enriched in each Ceratopteris segment. While most transcripts (19,533) are shared between all three tissues, we also detected sets of specific transcripts (Figure 1D). We found 1,071 genes preferentially expressed in the RT, 965 specifics for DR and 793 for SH. Interestingly, we detected 1,978 genes expressed simultaneously in both RT and SH, but not in DR, indicating that meristematic genes could be detected in both apical tissues, even if the SAM was only a small portion of complex SH sample.

**Figure 2:**
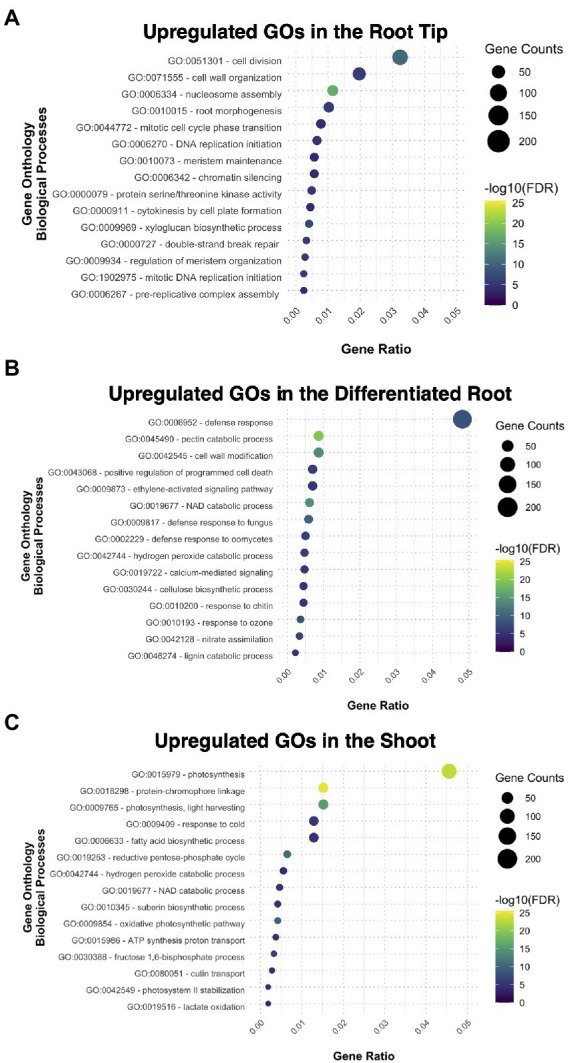
Gene ontology analysis exhibiting the different biological processes upregulated and enriched in each tissue: **(A)** the root tip, **(B)** the differentiated root, and **(C)** the shoot.

We performed gene ontology (GO) enrichment analysis for the sets of genes that are preferentially expressed in each of the three samples, genes that had a fold change (FC) value ≥2 compared to the other two segments. The root tip showed enrichment in categories related to cell division and root morphogenesis (Figure 2A); while other specific categories were related to the auxin transport and procambium development (Supplementary Figure 2A). This data suggest that the transcripts related to meristematic activity, cell cycle progression, and tissue histogenesis, are upregulated in the RT. DNA binding, transcription factor activity, and DNA replication were enriched categories for the molecular function (Supplementary Figure 2B), indicating that the RT bears a mitotically active meristematic region.

The cellular components ontology enriched in the root tip included the nucleosome and other structural components of cellular division in plants, such as the phragmoplast (Supplementary Figure 2C). The cell wall was also another GO category present in RT, together with plasmodesma and extracellular region (Supplementary Figure 2C); some other biological processes comprised biosynthetic and modeling processes of the cell wall (Supplementary Figure 2A). All together, these results imply the importance of the cell wall during root development since new cell walls are formed after each cell division.

In the case of DR, most enriched GO categories are related to tissue maturation, defense responses, nitrate assimilation, and ion transport (Figure 2B; Supplementary Figures 3A–C), reflecting some of the canonical functions for nutrient uptake in the root. The DEGs from SH were enriched for GO categories related to photosynthesis and catabolic processes, and functional categories related to developmental processes, such as guard cell fate commitment, vascular tissue patterning, and phloem transport (Figure 2C; Supplementary Figures 3A–C). Overall, our GO analysis suggests that each fragment of the young sporophyte have specific expressed genes related to their main functions in the plant body of other plant species, suggesting functional conservation.

### Expression of transcription factors families in *Ceratopteris* young sporophyte

**Figure 3:**
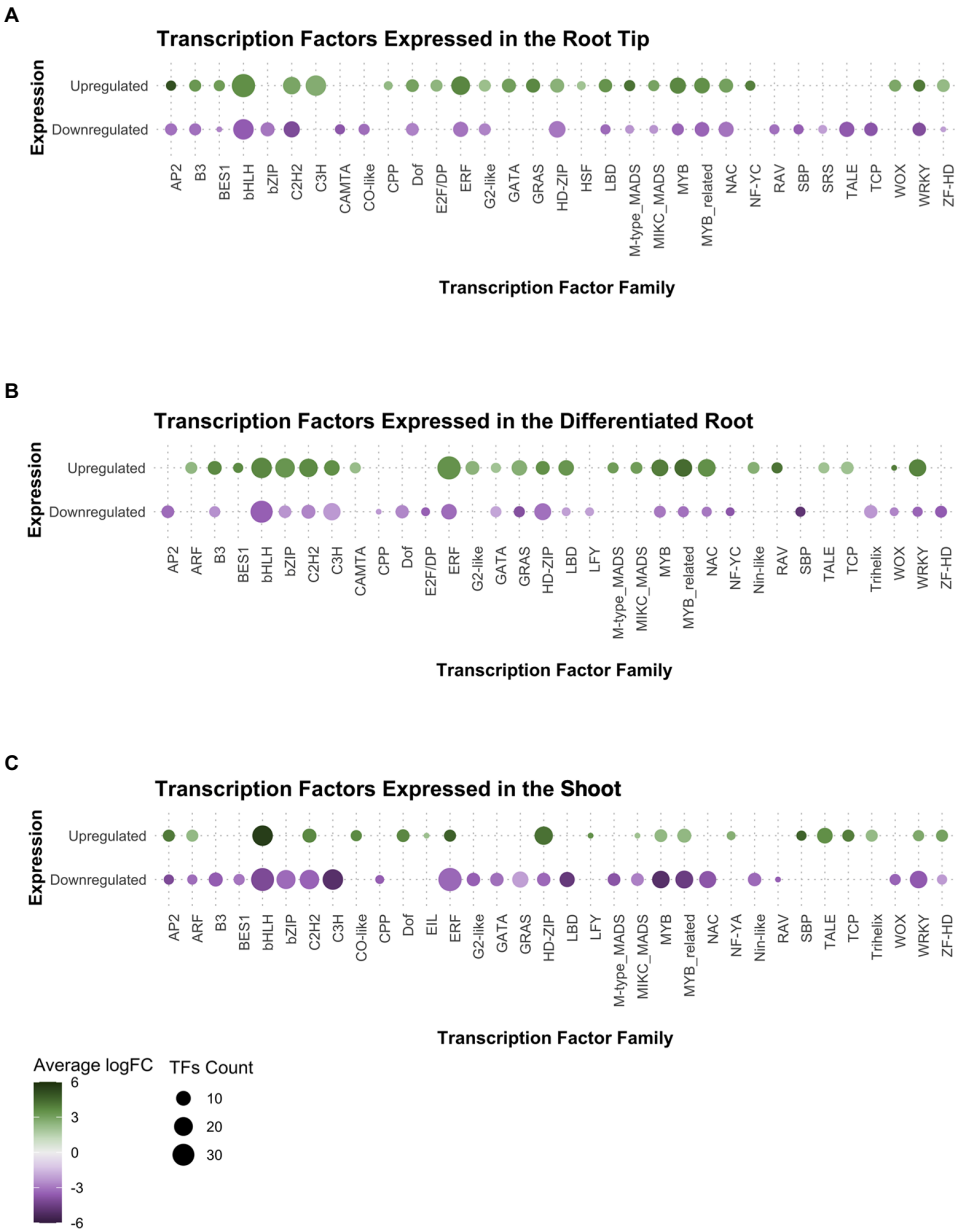
Analysis of gene expression in the transcription factor families found in Ceratopteris transcriptome per tissue: **(A)** the root tip, **(B)** the differentiated root, and **(C)** the shoot.

After observing that the transcription factors (TF) category was enriched in our GO analysis for the root tip, we decided to explore which TF families were preferentially expressed in the root meristem compared to DR and SH. We found that members of the following TF families were upregulated in the root tip (Figure 3A): (1) cysteine-rich polycomb-like protein (*CPP*) family, which includes the gene *TSO1* associated with meristem organization and cell division processes in the late inflorescence meristem of Arabidopsis (Liu et al., 1997; Song et al., 2000); (2) *E2F/DP* family, involved in the cell cycle regulation during the G1/S transition (de Almeida Engler et al., 2009; Sánchez-Camargo et al., 2021); (3) heat-shock factor (*HSF*) family, which incorporates *SCHIZORIZA* that has functions related to stem cell maintenance and cell proliferation in Arabidopsis root meristem (Pernas et al., 2010; Begum et al., 2013); and (4) Nuclear Factors Y type C (*NF-YC*), associated to different functions including meristem development (Laloum et al., 2013; Petroni et al., 2013; Myers et al., 2016).

Other TF families showed a negative FC in in RT while a positive FC in the other plant segments. That was the case for basic leucine zipper (*bZIP*), Calmodulin-binding Transcription Activator (*CAMTA*), and Related to ABI3 & VP1 (*RAV*) families in the differentiated root (Figure 3B), while *CONSTANS-like* and *SQUAMOSA*-promoter binding-like (*SBP*) families were presented in shoot (Figure 3C). Interestingly, Ethylene Insensitive3-Like (*EIL*) and LEAFY (*LFY*) genes were exclusively expressed in the shoot.

We searched for well-known transcription factors that play roles as plant developmental regulators. The *WUSCHEL homeobox-containing* (*WOX*) TF family, associated with stem cell maintenance, was expressed in both root tissues but preferentially at the tip (Figures 3A,B). Members of the *AP2* family, where the *ANT/PLT* genes are included, were found with positive fold change in the root tip and the shoot. We detected the *B3*, *BES1*, *LBD*, *NAC*, and *GRAS* gene families expressed only in both root segments. From those families, specific members of both NAC and GRAS proteins have been associated with different root developmental processes in flowering plants, including ground tissue development, root cap maturation, and vascular tissue differentiation (Scheres et al., 1995; Helariutta et al., 2000; Sabatini et al., 2003; Willemsen et al., 2008; Bennett et al., 2010; Cruz-Ramírez et al., 2012; Kumpf and Nowack, 2015). A close analysis for each family would allow to understand if the components associated to genetic networks and developmental mechanisms have been conserved between ferns and seed plants.

### *WOX* genes are expressed in the root meristem even in the absence of quiescence center

**Figure 4:**
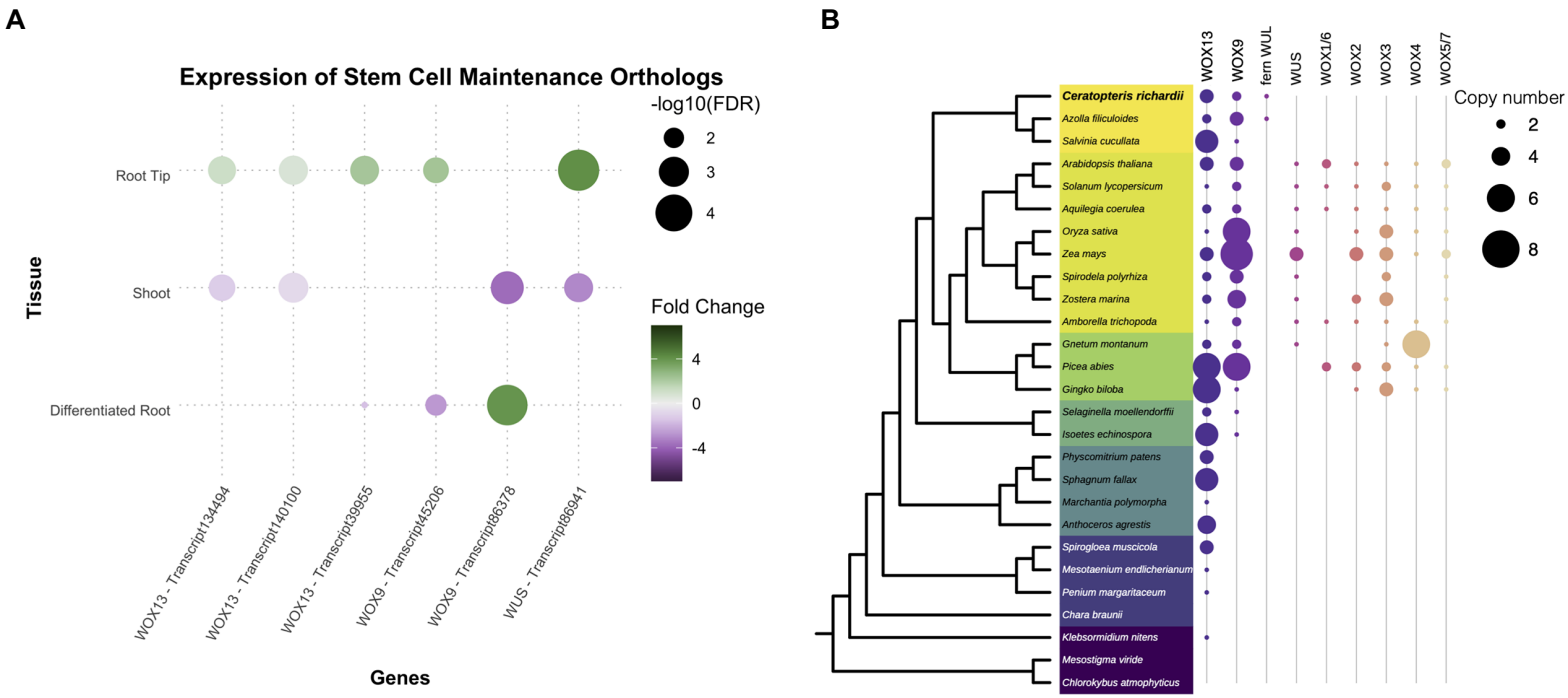
Conservation of genes involved in stem cell maintenance. **(A)** Expression of Ceratopteris ortholog *WUSCHEL homeobox-containing* (WOX) genes in the different segments. **(B)** Schematic representation of the presence and absence of orthologs for the different WOX clades throughout plant phylogeny.

The WOX genes are known to be part of the mechanisms involved in the maintenance of the stem cells and development of plant organs. Therefore, we decided to analyze in detail the different members of the *WOX* family since the family was expressing in Ceratopteris root. The *WOX* genes are classified into three clades: *WOX13*, *WOX9*, and *WUS* clades (Nardmann and Werr, 2012, 2013). We found six different transcripts corresponding to this family in the Ceratopteris transcriptome. Five of them were found preferentially expressed in RT, and one was more abundant in DR (Figure 4A). We continued by exploring the evolutionary history of this family in relation to ferns (Supplementary Figures 5–7). The WOX13 clade contains WOX10 and WOX13/14 from Arabidopsis (Supplementary Figure 5). We found three possible subgroups of this clade in ferns. The WOX9 clade includes two different groups from seed plants (Supplementary Figure 6): the WOX8/9 subclade, involved in root development during embryogenesis and the WOX11/12 subclade implicated in adventitious root initiation (Wu et al., 2005; Ueda et al., 2011; Yu et al., 2020). The WOX9 clade is only present in vascular plants (Figure 4B). In ferns, we found a single node comprising all members from the WOX9 clade. Ceratopteris *WOX* genes orthologs for *WOX9* have been previously name *CriWOXA* and *CriWOXB* (Nardmann and Werr, 2012).

Finally, the WUS-related clade was only found in the euphyllophytes (Figure 4B; Supplementary Figure 7). Putative members of this clade were found in Ceratopteris and Azolla, but not in Salvinia. In the seed plants, this clade has been subjected to different rounds of duplication as we detected six different subgroups. The fern WUSCHEL-like proteins (WUL) and all the diverse sets of WUS-related proteins from seed plants may be an innovation of the euphyllophytes. *CriWUL* was one of the *WOX* genes that were expressed in the root tip of Ceratopteris, implying its possible role in the root stem cell niche. Further functional analyses are needed to dissect and understand how all the different *WOX* genes function in root development and how they behave in other lineages besides flowering plants.

### Presence of the gene circuit components for the ground tissue specification

**Figure 5:**
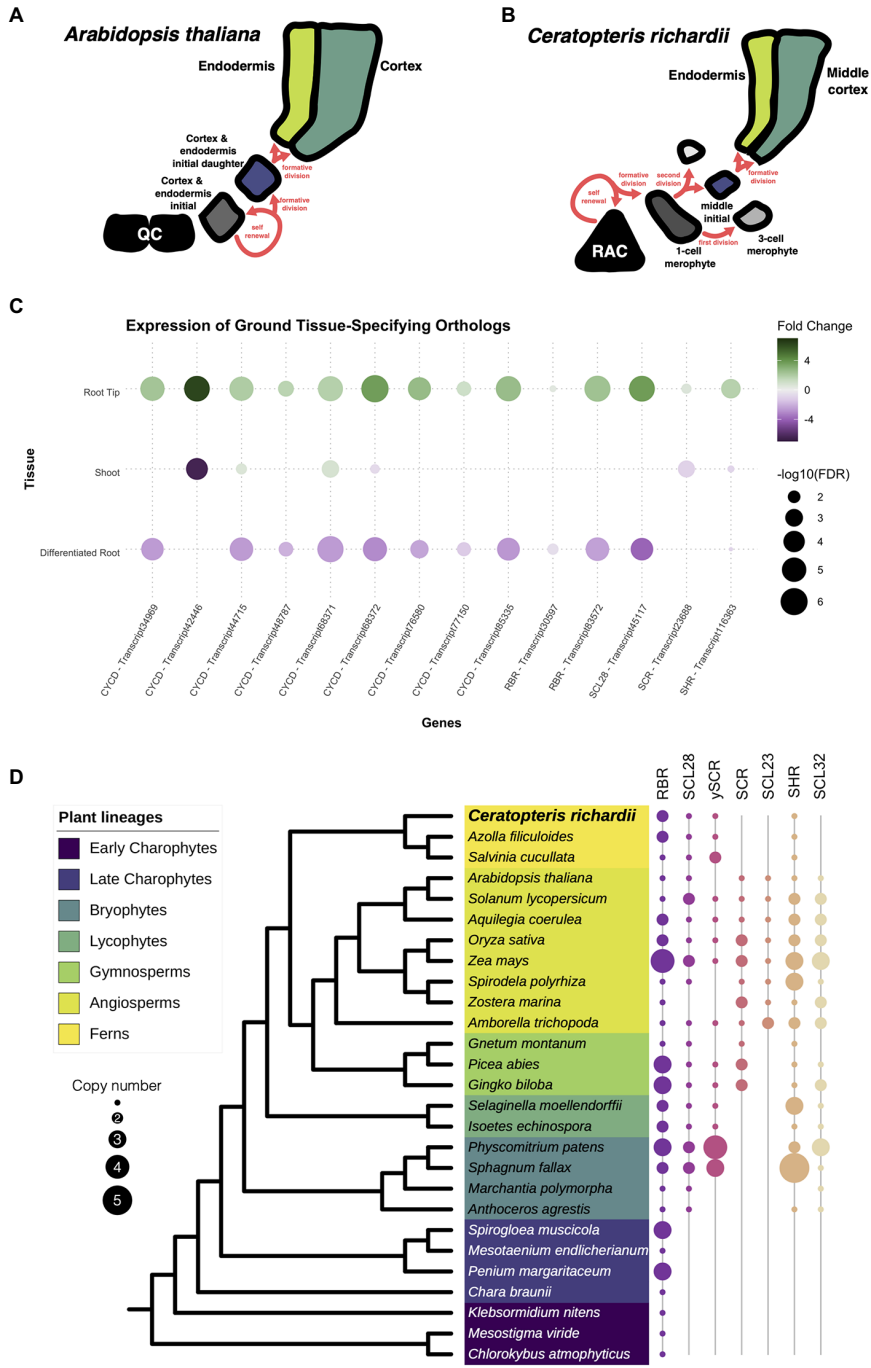
Conservation of genes involved in the specification of the ground tissue. **(A,B)** Schematic representation of the development of the cortex and endodermis between Arabidopsis and Ceratopteris. **(C)** Expression of Ceratopteris ortholog genes implicated in the cortex-endodermis specification in the different segments. **(D)** Schematic representation of the presence and absence of orthologs for RBR, SCR, and SHR throughout plant phylogeny.

To determine whether root developmental programs present in other plant species are also present Ceratopteris, we searched for a group of genes known to be involved in ground tissue development. In Arabidopsis, the endodermis and cortex are specified during a series of asymmetric cell divisions that begin from the cortex-endodermis initial (CEI; Figure 5A). In Ceratopteris, part of the ground tissue comes from the division of the merophyte middle initial that generates the endodermis and the middle cortex (Figure 5B). We asked if the different genes involved in ground tissue specification were conserved among Ceratopteris and Arabidopsis. A complex gene circuit has been associated with the specification of the cortex and endodermis, in which AthSHORTROOT (AthSHR), AthSCARECROW (AthSCR), AthRETINOBLASTOMA-RELATED (AthRBR), and AthCYCLIN D6;1 (AthCYCD6;1) participate (Cruz-Ramírez et al., 2012). In the expression data from Ceratopteris, we found that possible orthologs of the *CYCD* family were expressed in the root tip, which agree with our previous findings of highly mitotic-active cells in the root meristem. We also identified two different transcripts for the *RBR*, which display different expression patterns in the root tip, while orthologs for the GRAS genes *AthSCR* and *AthSHR* were also present in the transcriptome and were differently upregulated in the RT compared to the segments.

Then, we investigated the evolutionary trajectory of the proteins encoded by some of genes involved in ground tissue specification. We assessed the evolution of the complete CYCD family, since CYCD6;1 belongs to this large family without a current well-supported phylogenetic reconstruction (Menges et al., 2007). Although we found CYCD proteins in the early-divergent charophyte algae, we did not recover any member from *Chara braunii* or algae from the class Zygnematophyaceae. In the embryophytes, the CYCD proteins displayed an interesting evolutionary history with two main clades. The first clade was present within every analyzed lineage, including AthCYCD7. In this clade, we discovered four different copies in the Ceratopteris transcriptome (Supplementary Figure 8). In the case of the second clade, this group displayed a complex evolutionary history. This CYCD lineage was only present in the euphyllophytes, and in ferns we found three different subgroups (CYCD-F1 to F3; Supplementary Figure 8), while five subgroups are present in seed plants (CYCD1-3,5 and 6; Supplementary Figure 9).

The evolutionary history of RBR was rather straightforward (Supplementary Figure 10). At least one copy is present in all analyzed lineages, from chlorophyte algae to streptophytes (Figure 5D). While extra copies were detected in certain species, we only found two consistent lineage-specific duplications, in ferns and monocotyledons. The duplication in ferns arises after the split from Equisetales because RBR sequences from the horsetail *Equisetum hyemale* were recovered adjacent to the duplication event. We recovered sequences from both fern RBR clades (Supplementary Figure 10), only one of these copies was present in all ferns analyzed (fern RBR1), while the other copy (fern RBR2) was present in the Azolla and Salvinia genomes, and the transcriptome of Ceratopteris, but absent in the majority of OneKP transcriptomes from ferns. Interestingly, the transcripts of *RBR2* display an elevated fold change in our RT dataset compared to the DR and SH (Figure 5C). The expression of this gene in the meristematic tissue could suggest that RBR2 could be carrying out specific functions such as modulating cell division or stem cell maintenance, as it does in Arabidopsis. While *RBR1* transcripts were still present in the root tip, their expression levels did not drastically change between tissues.

A previous study has approached the evolution of the whole GRAS family while finding interesting gene expansions and losses over the Embryphyte linage (Geng et al., 2021). In the case of this family, we decided to focus only on the evolution of those GRAS proteins involved in the cortex and endodermis specification: SCR (including SCL28 & SCL23) and SHR (including SCL32). The phylogenetic reconstruction for SCR revealed an intriguing evolutionary history (Figure 5D; Supplementary Figure 11). We also reconstructed the phylogeny of SCL28 due to its closeness to SCR in a previous phylogenetic reconstruction and function in the root meristem in Arabidopsis (Lee et al., 2008; Goldy et al., 2021). We found that orthologs for SCR are present in almost all embryophyte lineages analyzed, except in the liverwort *Marchantia polymorpha*. While we also did not detect a copy in *Anthoceros agrestis*, a SCR ortholog was present in *Anthoceros augustus* (Supplementary Figure 11). Also, several expansion events occurred in seed plants; the first duplication appears to have taken place at the base of seed plants. While our data suggest the appearance of the SCR lineage and another SCR-like lineage, which orthologs are not present in all flowering plants. The second duplication gave rise to the appearance of SCL23 at the base of the angiosperms. In the case of ferns, the protein sequences of this lineage clustered together with the SCR-like clade (Supplementary Figure 11). For that reason, we denominated this copy as the SCR pro-ortholog (γSCR). Besides that, the SCR copy of Ceratopteris was expressed in the root tip, indicating a possible function in ground tissue specification and/or stem cell maintenance (Figure 5C).

Additionally, we examine the evolution of SHR in embryophytes (Figure 5D; Supplementary Figure 12). This GRAS protein was found in all the different species analyzed, except to *M. polymorpha*. In the case of ferns, a single copy of SHR was present in all analyzed species, and this clade predates the duplication in flowering plants. The transcript of *CriSHR* was found differentially expressed in the root tip compared to the other fragments of Ceratopteris. We also noticed that SCL32, the closest relative to SHR, was not present in any analyzed fern, but it is present in the other vascular plant lineages. The absence of SCL32 in ferns suggests lineage-specific gene loss, which has been previously found in a recent study (Geng et al., 2021). Interestingly, a similar absence of orthologs for SCR and SHR in *M. polymorpha* has been also reported in the phylogenetic reconstruction of HAM, another GRAS protein (Geng et al., 2021). This consistent loss of major developmental regulators could be related to the reductive evolution that has been reported in liverworts, i.e., the loss of genes involved in stomata developmet (Harris et al., 2020).

### Unique evolutionary histories for the components of the root cap specification

**Figure 6:**
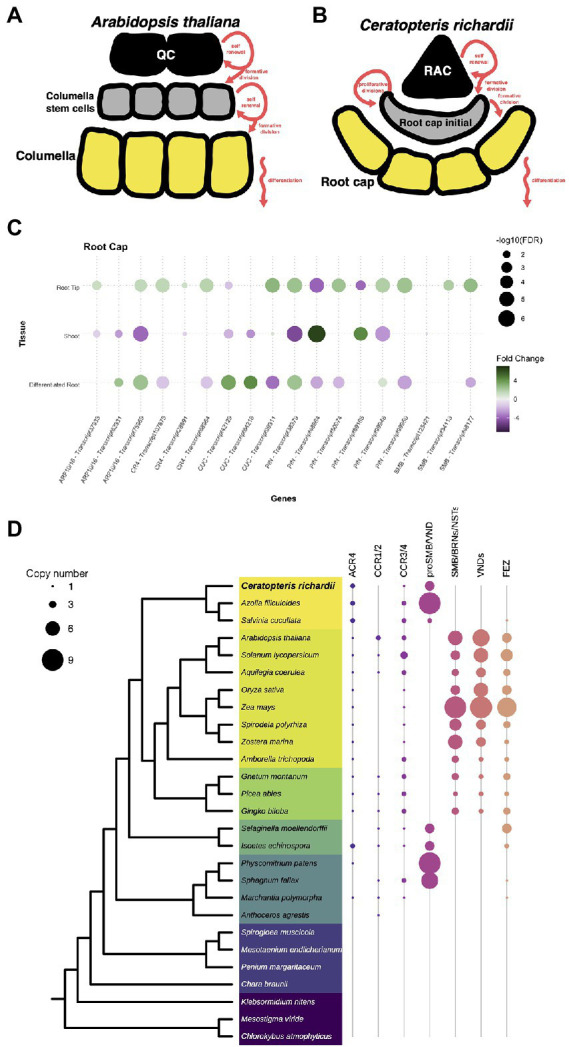
Conservation of genes involved in the specification of the root cap. **(A,B)** Schematic representation of the development of root cap between Arabidopsis and Ceratopteris. **(C)** Expression of Ceratopteris ortholog genes implicated in the root cap development and maturation in the segments tissues. **(D)** Schematic representation of the presence and absence of orthologs for ACR4, SMB, and FEZ throughout plant phylogeny.

We also analyzed genes involved in the development and maturation of the root cap as this structure was a new acquisition during the evolution of roots. In Arabidopsis, the root cap cell is divided the columella and the lateral root cap. The columnella which is populated by the asymmetric division of distal stem cells in the root meristem (Figure 6A). In Ceratopteris, there is no specification of the collumella and lateral root cap cells but the generation of the root cap initial (RCI) resembles the first step of columella specification (Figure 6B). The following steps in Ceratopteris root cap maturation are specific from this species since the RCI follows a series of proliferative divisions. Despite the different organization and development of the root cap in these distant plant lineages, we decided to analyze if the orthologous regulatory genes are present in Ceratopteris that could suggest a similar in the mechanism governing the root cap maturation.

First, we looked for orthologs of genes related to auxin response and transport, the ARF10/16 and the PIN3/7 proteins. We identified three putative *ARF10/16* orthologs, which were differentially expressed in both root tissues compared to the SH (Figure 6C). One of them was exclusively expressed in the root tip. In addition, we detected six *PIN* orthologs expressed in at least one of Ceratopteris segments. Two *PIN* genes were found only expressed in the root tip. We did not evaluate the gene evolution of these families since it has been previously assessed (Bennett et al., 2014; Mutte et al., 2018).

We also found three putative orthologs for *ACR4*. All of them were more highly expressed in RT compared to the other tissues (Figure 6C). Phylogenetic reconstruction allowed the identification of two orthologs for *ACR4* in Ceratopteris, and a third member more related to the *CCR3/4* clade (Figure 6D). In the case of the two ACR4 orthologs, the phylogenetic tree demonstrated that there is gene duplication in the fern lineage (Supplementary Figure 13).

In the case of the NAC protein family, we identified 29 transcripts that contained a NAC domain, but we decided to focus only on SMB and FEZ that are related to root cap development. We identified four putative orthologs for *SMB* and their closest relatives. Two of these sequences had higher levels in RT than in DR and SH, in concordance with their possible role in the Ceratopteris root cap development (Figure 6C). We generated a phylogenetic tree to evaluate the evolutionary history of these proteins (Figure 6D; Supplementary Figure 14). In euphyllophytes, the data suggest that each lineage had an intricate evolutionary history after the split between ferns and seed plants. Several rounds of duplication took place in each plant clade. In the case of seed plants, these duplications gave rise to two main protein subgroups: (1) SMB together with BRNs and NSTs; (2) all different VNDs proteins. We detected four subgroups in ferns but still without known developmental functions; Ceratopteris transcripts were present only in three groups. Because two of them are expressed in the root apical meristem, we suggest that their involvement in root development predates the separation of euphyllophytes.

We did not find any sequence related to *FEZ* in the Ceratopteris transcriptome, nor in the current version of the genome. Still, we assessed the evolutionary history of this protein (Supplementary Figure 15). The phylogenetic reconstruction of FEZ showed no orthologs of this TF neither in Ceratopteris nor in Azolla (Figure 6D). Nevertheless, we detected a possible copy of FEZ in Salvinia (Supplementary Figure 15); this was interesting due to the fact that Salvinia does not develop a root cap. We also found FEZ orthologs in other ferns and lycophytes, including *Selaginella moellendorffii* and *Isoëtes echisnospora*; the data suggest that this well-known root cap regulator was lost in Ceratopteris. We found three putative transcripts in the case of the *CUC* genes; two were expressed in the differentiated root (Figure 5C). The other one was upregulated in the root tip and slightly expressed in the shoot organs, which may suggest that *CUC* expression could be overshadowed in the SH because the SAM was only a small portion of what we captured in this segment.

### Comparison with a public Ceratopteris RNA-seq dataset

While we were analyzing our RNA-seq data, another Ceratopteris transcriptome was published where they analyzed five different segments: stem (including SAM); leaves; leaf tips; roots; and root tips (Yu et al., 2020). We decided to take this recently available published dataset and compared it with our data (Yu et al., 2020). In the case of our dataset, we used the Hn-n strain since the reference genome was built for this cultivar., while the other RNA-seq dataset used RNWT1 strain. In addition, we selected root tips that belonged to root with active growth and dissect the root segments based on the different developmental stages. Comparison of two different RNA-seq dataset would allow to detect possible differences in gene expression due to different and specific sporophyte organs and to enrich transcriptional analysis in Ceratopteris sporophyte.

**Figure 7:**
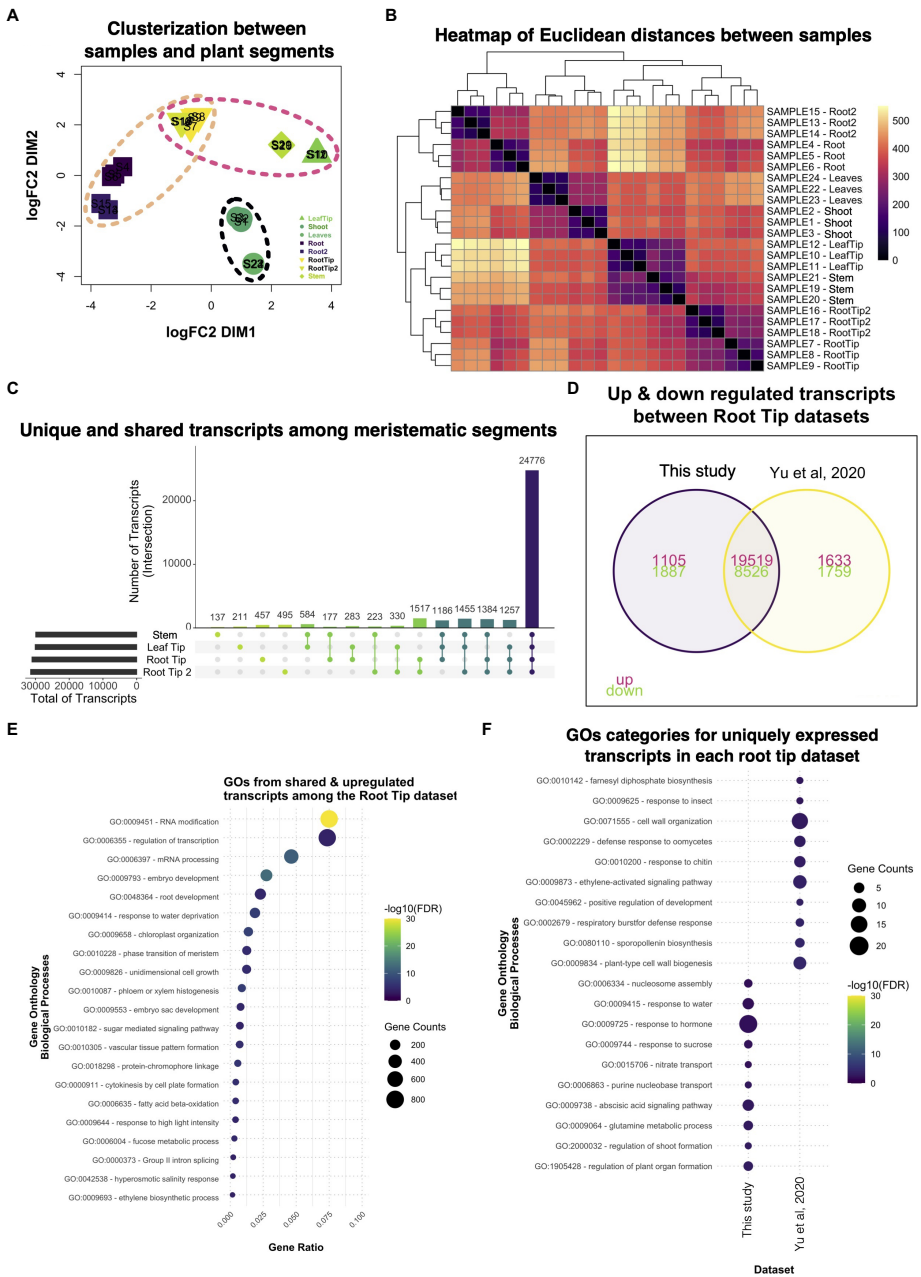
Comparative analysis between two different datasets of Ceratopteris sporophytic tissues. **(A)** Multidimensional scaling analysis to examine how similar and contrasting are the samples and plant parts of both datasets. **(B)** Heatmap showing the relatedness between samples and plant segments in the two datasets. **(C)** Plot showing the unique and shared transcripts between meristematic segments. **(D)** Venn diagram exhibiting how many transcripts are shared or unique among root tip datasets. **(E)** GO enrichment analysis of the shared transcripts between both root tips samples. **(F)** GO enrichment analysis of transcripts that are expressed uniquely in each root tip dataset.

Multidimensional scaling analysis detected that the root tip datasets were arranged closely (Figure 7A), but separated from the other meristematic tissues, the stem and the leaf tip. It was interesting to notice that both root tips datasets distributed differently from the stem tissue in the MDS analysis since the root, as well as the leaves, have their origin from apical cell at the shoot apical meristem (Hou and Hill, 2002). The datasets from mature segments were also clustered together and separated from the meristematic tissues. These clustering patterns were also noticeable in the heatmap (Figure 7B), where the differences in mature parts are greater than between the meristematic tissues based on their positions of the cladogram.

We searched for genes expressed in each meristematic tissue since we wanted to know how many genes overlap between the tissues that harbor stem cells. From the public dataset, we found that the stem only had 137 specific genes; this segment also shared the most genes with the leaf tip in pairwise comparisons (Figure 7C). We decided to assess which GO categories were enriched for upregulated genes in all meristematic tissues (Supplementary Figure 16A). The GO terms fell into categories of regulation of gene expression, metabolic processes, and chromatin remodeling. Only a few GO terms were related to cell division processes. This could suggest that the diverse meristematic tissues do not harbor a similar population of mitotic-active cells or their stem cells, in the respective niches, do not divide as frequently as the root tip. One category that caught our attention was Embryogenesis, which indicates that meristematic tissues resemble the embryonic tissues during development.

Then, we decided to focus on the differences and similarities between the root tip dataset generated in our study and the public root tip dataset. We found that both datasets shared the largest number of genes (>1,500) in paired comparisons (Figure 7D). We also noticed that each dataset contained over 450 genes uniquely expressed even though they were the same type of segment. We identified GO terms related to gene transcription as the most enriched categories in shared transcripts of the root tip datasets (Figure 7E). Other important categories were developmental processes involved in the embryo, meristems, root, and vascular tissue formation, which includes mechanisms transcripts related to growth, pattern formation, and regulation. This data suggest that the root tips of both studies exhibit the transcriptomic fingerprint of an active meristematic tissue.

To detect differences between the datasets, we looked for GO terms for the uniquely expressed genes for each dataset (Figure 7F). Our RT dataset showed categories related to organ formation, response to hormones, and nucleosome modeling, which indicates a highly active and responsive root apical meristem. While Yu et al. (2020) dataset revealed enrichment in cell wall processes, development, and response to biotic stress. The appearance of different GO categories related to biotic stimuli in this dataset were like those found for the differentiated root (Figure 2B). Overall, both root tips datasets provided new insights into how the root apical meristem behaves at the molecular level in ferns and could be used in further studies to understand gene network conservation in the euphyllophytes roots.

Finally, since we detected a small number of genes exclusively expressed between the stem and the leaf tip of the public dataset, we determined GO enrichment for genes uniquely expressed in each of these segments. In the case of the leaf tip, we observed GO terms linked to hormone response, photosynthesis, and leaf development (Supplementary Figure 16B). For the stem, most GO terms were associated with the development of plant organs absent in ferns, such as anther, stigma, and style, suggesting that many regulators were co-opted for different developmental processes during plant evolution (Supplementary Figure 16B). This type of analysis will help understanding how genes behave in other plant lineages besides flowering plants.

## Discussion

### Ceratopteris root tip expresses genes conserved in vascular plants

Generation of transcriptomes from emerging model species encourages the assessment of the molecular mechanisms that evolved in these plants. Here, we report a *de novo* transcriptome assembly of *C. richardii*, a species that has been used as a fern model organism to understand the developmental processes in this plant lineage (Marchant, 2019). Studies incorporating transcriptomes for Ceratopteris were previously published from different organs, treatments, or developmental stages: spore germination, gametophyte development, and auxin response (Salmi et al., 2005; Bushart et al., 2013; Atallah et al., 2018; Mutte et al., 2018; Yu et al., 2020; Geng et al., 2021). While other transcriptome reports were focused in producing transcriptome assemblies for subsequent analyses, this is the first report where a Ceratopteris transcriptome has focused on a detailed analysis on gene expression in the root meristem and its comparisons against mature organs.

Each organ has its own transcriptional signature; therefore, performing differential gene expression analysis allowed the comparison of transcripts from different organs or organ segments that differ in their differentiation level and enables in future the analysis of the transcriptome changes upon development. This opens the possibility of detecting conserved gene circuits but also novel regulators for root development in this or related species in the fern lineage (Brady et al., 2006). Even though we only generated data from plants in optimal growth conditions, this dataset could be later used as default developmental stages.

Transcriptome analysis showed differences between the GO categories enriched for each tissue. RT showed enrichment in categories akin to organ development, meristem maintenance, and cell cycle processes, which are consistently associated to an active meristem. Related categories have been found in the meristematic regions of other plants. In Arabidopsis roots, sections that include the QC and the stem cell niche displayed GOs enriched in meristem determinacy and mitosis (Brady et al., 2007). Categories for DNA replication and cell cycle were detected in the RAM of *Oryza sativa* during a transcriptional analysis. Also, the presence of the cell wall organization, xyloglucan biosynthesis, and ethylene response categories in Ceratopteris root tip suggests that we captured the development processes of the root cap, as it was reported previously for the rice root cap (Takehisa et al., 2012).

Comparative analysis of Arabidopsis, rice and tomato, showed that genes related to cell division, cell wall biosynthesis, and transcriptional regulation were preferentially expressed in the root meristem, which suggests a conservation of a fundamental program for angiosperms’ root meristem (Kajala et al., 2021). Our results of Ceratopteris root meristem expression share similar functions found to be important in the development of the root meristem in flowering plants. Previous studies including meristematic root tissues from *Selaginella moellendorffii* showed that gene expression is conserved between orthologs of vascular plants. Root meristem genes predate the appearance of roots in tracheophytes, suggesting that these genes and their networks were co-opted from other developmental process (Huang and Schiefelbein, 2015; Ferrari et al., 2020). Based on the previous statements, we postulate that both vascular plant lineages, lycophytes, and euphyllophytes, may have arrived at the same outcome by independently co-opting a similar set of genes during root evolution.

### Transcription factors important for plant development have a conserved expression in Ceratopteris

One of the features in our data was that hormone response regulators for auxin (ARF) or cytokinin (ARR) did not showed a significant fold change in the root tip compared to other plant segments based on our cut-off of FC > 2. However, we found that ARF genes are preferentially expressed in the differentiated root, as well as in the shoot (Figure 3A). Other TF families were identified to be preferentially expressed in the root tip. One of those was the specific expression of the members E2F/DP gene family in Ceratopteris root tip, which confirms the proliferative state of this tissue. In Arabidopsis, these genes have high expression in the root meristem. Other genes involved in cell cycle progression were reported to be also expressed in Arabidopsis root tip, such as CDKs and CYCLINs (de Almeida Engler et al., 2009; Sánchez-Camargo et al., 2021). Further studies will be required to test if the expression of RBR orthologs in the Ceratopteris root tip implies the direct interaction with CDKs to regulate the G1-S checkpoint in the cell cycle.

The *LEAFY* (*LFY*) genes were found exclusively expressed in the shoot. This data are concordant with a previous report in Ceratopteris, where *LFY* presented a species-specific duplication and at least one of the copies was found expressed only in the SAM and leaves (Plackett et al., 2018). The preferential expression of specific transcription factors between the different organs of Ceratopteris indicates an intricate network of interactions that would generate a unique repertoire of target genes. These differences would produce the proper developmental processes that shape the formation of a complete plant body.

While there are several genes involved in stem cell maintenance in the apical meristems of flowering plants, we decided to focus on the *WOX* genes since they were expressed in the root tip of Ceratopteris (Figure 3A). A few reports have been published about their presence or function in other vascular plants (Nardmann and Werr, 2012; Ge et al., 2016; Youngstrom et al., 2019). In Ceratopteris RAM, we detected the expression of members from the three different *WOX* lineages. The genes *CriWOXA* (transcript45206; *WOX9* clade) and *CriWUL* (transcript86941; *WUS* clade) were upregulated in the root tip dataset compared to the other segments. This result coincides with a previous study where the expression of both genes was detected in Ceratopteris root tip by *in situ* hybridization assays (Nardmann and Werr, 2012). The function of these genes in Ceratopteris root still remains unanswered. But, both genes are upregulated in ectopic root primordia of Ceratopteris after auxin treatment, which suggests a possible role in root initiation (Yu et al., 2020).

Moreover, *CriWOXB* (transcript86378; *WOX9* clade) was only differentially expressed in the differentiated root. The result contrasts with previous reports since it was also found in the root tip and other proliferative tissues, such as leaf and shoot apical meristems (Nardmann and Werr, 2012; Youngstrom et al., 2019). This could be due to *CriWOXB* being mainly expressed during lateral root (LR) formation.

Both *CriWOXA* and *CriWOXB* genes belong to the *WOX9* clade of this gene family. Their orthologs are *WOX8* and *WOX9*, which are important for proper embryo development of different seed plants and for meristem maintenance in Arabidopsis (Wu et al., 2005; Breuninger et al., 2008; Ueda et al., 2011; Zhu et al., 2014). In Arabidopsis, *AthWOX8* and *AthWOX9* precede the expression of *AthWOX5* in the embryo. In Ceratopteris, *in vitro* assays have shown that CriWOXA activates the expression of *CriWUL*. This could indicate a conserved mechanism where *WOX9* genes activate *WUS* genes (Yu et al., 2020). It would be interesting to assess if this mechanism is conserved during Ceratopteris embryo development, mainly in the specification of the first root apical cell.

### The evolution of the CYCD-RBR-SCR-SHR network

The specification of the cortex and endodermis is one of the best characterized developmental processes in Arabidopsis. A set of essential players for this mechanism has been identified in different studies including the GRAS domain proteins SCR and SHR, and the cell cycle regulator RBR (Scheres et al., 1995; di Laurenzio et al., 1996; Helariutta et al., 2000; Cruz-Ramírez et al., 2012). Throughout phylogenetic reconstruction, we identified possible orthologous genes for the different members of this gene circuit. This is the first report, as far as we know, that has evaluated the evolution of CYCDs using members from all extant groups of streptophytes. A similar phylogenetic reconstruction was previously reported using three different angiosperms and one bryophyte, but it did not cover the whole plant diversity (Menges et al., 2007). Overall, we detected that CYCDs split into two main lineages, one only present in euphyllophytes following extensive rounds of duplication. Our data suggest that gene duplications of the second clade occurred independently in ferns and seed plants. This indicates that there is no direct ortholog for AthCYCD6;1 in Ceratopteris, but any of the CYCDs present in this fern could potentially function as part of the CYCD-CDK complexes that interact and phosphorylate RBR.

In ferns, the evolution of RBR suggests a lineage-specific duplication present in all lineages but Equisetales. While *RBR* is maintained as a single copy in many plant lineages and species, we can detect duplications that are species-specific. Duplication events of this gene that are consistent throughout a whole plant lineage are very rare. Only in monocots, a persistent duplication has been detected (Lendvai et al., 2007; Desvoyes et al., 2014). In maize, members from both lineages may have different roles. While *RBR1* is constitutively expressed, *RBR3* is only expressed during mitosis (Sabelli et al., 2005). Nevertheless, *CriRBR2* was highly expressed in the root tip compared to *CriRBR1*, which could indicate a divergence in the expression pattern or a possible function for meristematic or proliferative tissues.

Since the families of GRAS TFs contain several members in diverse studied plant species, the evolution of this family is rather complex. A previous report has untangled the evolution of all the different GRAS domain proteins by separating them in orthogroups or predicted (sub)gene families (Geng et al., 2021). As similar as our approach, both SCR and SHR were found in individual orthogroups. Moreover, we found that SCR arose from duplication, possibly at the base of seed plants. The γSCR, the other product of this duplication was found in ferns, gymnosperms, and only a few angiosperms. The *γSCR* from tomato was observed highly expressed during arbuscular mycorrhizal development and this gene may play an important role in controlling arbuscular colonization (Ho-Plágaro et al., 2019). In *Medicago truncatula*, a similar function was found, since this gene is an important regulator of nodulation during legume-rhizobium symbiosis (Kim and Nam, 2013). While reconstructing the phylogeny of SCR closest relatives, we did not find any evidence of a *SCL23* copy. In fact, this duplication event occurred at the base of angiosperms; this copy is considered the closest relative to SCR and has also a function in the ground tissue of Arabidopsis (Long et al., 2015). Besides, we discovered the presence of a possible ortholog for *AthSCL28*, which was also found to be differentially expressed at the root tip. AthSCL28 has an important role during the mitotic phase at Arabidopsis root meristem (Goldy et al., 2021).

For SHR, we also detected a possible duplication event in the common ancestor of angiosperms, but this SHR-like copy was not present in all the species analyzed. In tomato, this *SHR-like* gene has been associated with cell proliferation during leaf development and was slightly upregulated during arbuscular mycorrhizal development (Coneva et al., 2017; Ho-Plágaro et al., 2019). For the possible *SHR* orthologs in ferns, this gene was present in this plant lineage including Ceratopteris. The *SHR* ortholog gene was also positively expressed in Ceratopteris root tip, suggesting a possible role in root meristem development. *SHR* function has been related to radial patterning and cell fate specification (Scheres et al., 1995; Helariutta et al., 2000; Nakajima et al., 2001). The symplastic movement of SHR is conserved among several angiosperms and even expanded to other cell layers in the root; nevertheless, the assessment of CriSHR protein moving through Ceratopteris root tissues would be interesting since a high number of plasmodesma connections have been detected in the root meristems of ferns. In addition, the conservation of CriSCR-CriSHR interaction needs to be tested in order to understand if their physical interaction has been preserve during evolution (Cui et al., 2007; Henry et al., 2017; Imaichi et al., 2018; Xiao et al., 2019; Dong et al., 2021; Kajala et al., 2021). In general, the conservation of all these genes, i.e., SCR, SHR, and RBR, and their expression in the root tip, may indicate a similar role of this whole gene circuit as observed in seed plants.

### Differences between different root tip expression datasets

We also compared our transcriptome datasets with recently published RNA-seq samples from five different segments of Ceratopteris (Yu et al., 2020). The published dataset came from a study aimed at studying the function of auxin during root initiation; and it gave us the opportunity to compare the dataset and detect consistency of gene expression between tissues. Overall, both root tip datasets grouped together, but we detected certain transcripts that were present in only one of the datasets. Some of the reasons related to these differences could be due to the actual longitudinal position from where the root tip explant was excised, that samples were in different developmental stages at the time of tissue harvesting or the confounding effects generated from different laboratories (Kajala et al., 2021). From transcripts present only in the Yu et al. (2020) dataset, we found GO categories associated with the mature root such as ethylene response and defense response; this could suggest that probably this segment was already entering a meristem termination program.

An interesting category in the public dataset was related to the biosynthesis of sporopollenin, and while this process is carried out during spore development, it may share some enzymes with the biosynthesis of aromatic compounds or acyl lipids associated with differentiation like suberin and lignin (Wang et al., 2018). The presence of these categories in the root tip could imply the exhaustion of the RAM, a process that we previously observed indirectly during a Ceratopteris root growth analysis (Aragón-Raygoza et al., 2020). Actually, a previous report indicates that the metabolism of aromatic amino acids is enriched during the terminal developmental stage of the root meristem in the cacti *Pachycereus pringlei* and resembles the differentiation zone of Arabidopsis root (Rodriguez-Alonso et al., 2018). While the root meristems of both organisms have different structures, the mechanisms relying on meristem termination or tissue differentiation could be conserved. Still, both root tip dataset shared functions related to cell division and transcription regulation which indicates meristematic activity.

There is a growing interest in exploring the evolution of plants throughout their diversity. Gene expression data presented, such as the one reported in this work for Ceratopteris, represents a useful resource to assess transcriptional programs in ferns and to better understand the molecular adaptations for this lineage. The characterization of transcriptional signatures of organs and developmental transitions is providing important knowledge to study plant biology with insights into the evolution of the plant body. These new data is being valuable to identify conserved gene modules that are expressed in the root apical meristem across euphyllophytes, as we presented two different examples of genes involved in different roles of root development in Arabidopsis, from the ground tissue specification and root cap maturation. However, further functional genetic analyses are needed in order to uncover the role of all these different genes. Similar analyses for other parts of Ceratopteris such as the embryo or fertile leaves will allow understanding the differences of these structures that differ greatly from other lineages. Also, they will allow deciphering the different paths that plants have followed during their evolution to persist on the current ecosystems.

## Data availability statement

*C. richardii* RNA-seq data generated for this study can be found in NCBI GEO repository under the accession number GSE207873.

## Author contributions

AA-R and AC-R conceived the project. AA-R, AC-R, and LH-E designed the experiments. AC-R supervised the project. AA-R performed the experiments, and analyzed the data and wrote the manuscript. All authors helped in the interpretation of results and revised the manuscript. All authors contributed to the article and approved the submitted version.

## Funding

AA-R was financially supported by a CONACyT PhD Fellowship (No. 421596).

## Conflict of interest

The authors declare that the research was conducted in the absence of any commercial or financial relationships that could be construed as a potential conflict of interest.

## Publisher’s note

All claims expressed in this article are solely those of the authors and do not necessarily represent those of their affiliated organizations, or those of the publisher, the editors and the reviewers. Any product that may be evaluated in this article, or claim that may be made by its manufacturer, is not guaranteed or endorsed by the publisher.

## Footnotes

^1^ https://github.com/TransDecoder/TransDecoder/wiki

^2^ https://mafft.cbrc.jp/alignment/server/
