# Supplementary material for: Transcriptional analysis of Ceratopteris richardii young sporophyte reveals conservation of stem cell factors in the root apical meristem
Source: Front Plant Sci. 2022 Aug 11;13:924660. doi: 10.3389/fpls.2022.924660 (PMC9413220; doi:10.3389/fpls.2022.924660)
Supplement: Supplementary file 1 [file Presentation_1.pdf]

frontiers

Supplementary Material

1 Supplementary Figures and Tables

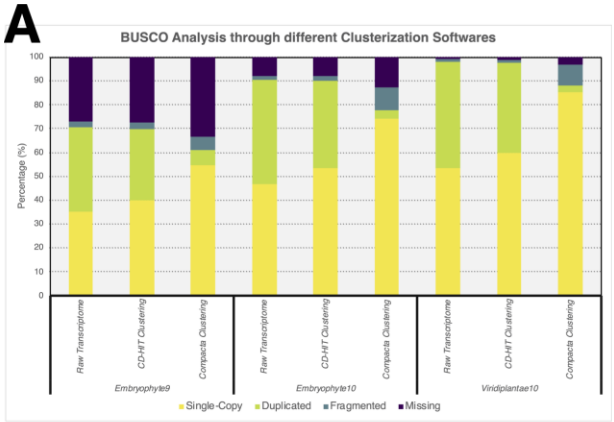

B

Ceratopteris richardii Hn-n	
**Transcriptome Size Comparison**	
Mutte et al., 2108	57,950
Marchant et al., 2019	18,179
Yu et al., 2020	88,641
Genome v2 from JGI	75,235
This study	154,546

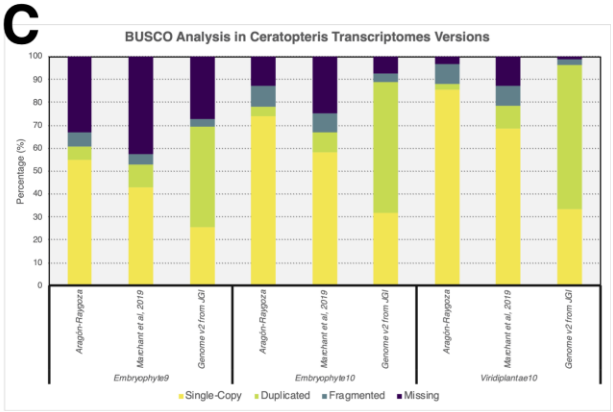

Supplementary Figure 1. Assessment of the transcriptome completeness. (**A**) Comparison of gene content during the different steps of transcriptome clusterization while using all the BUSCO databases for plants. (**B**) Table comparing the transcripts number between reported Ceratopteris transcriptomes. (**C**) Comparison of gene content between different versions of the Ceratopteris predicted transcriptome (from the genome) and the *de novo* transcriptome using the BUSCO Plants databases.

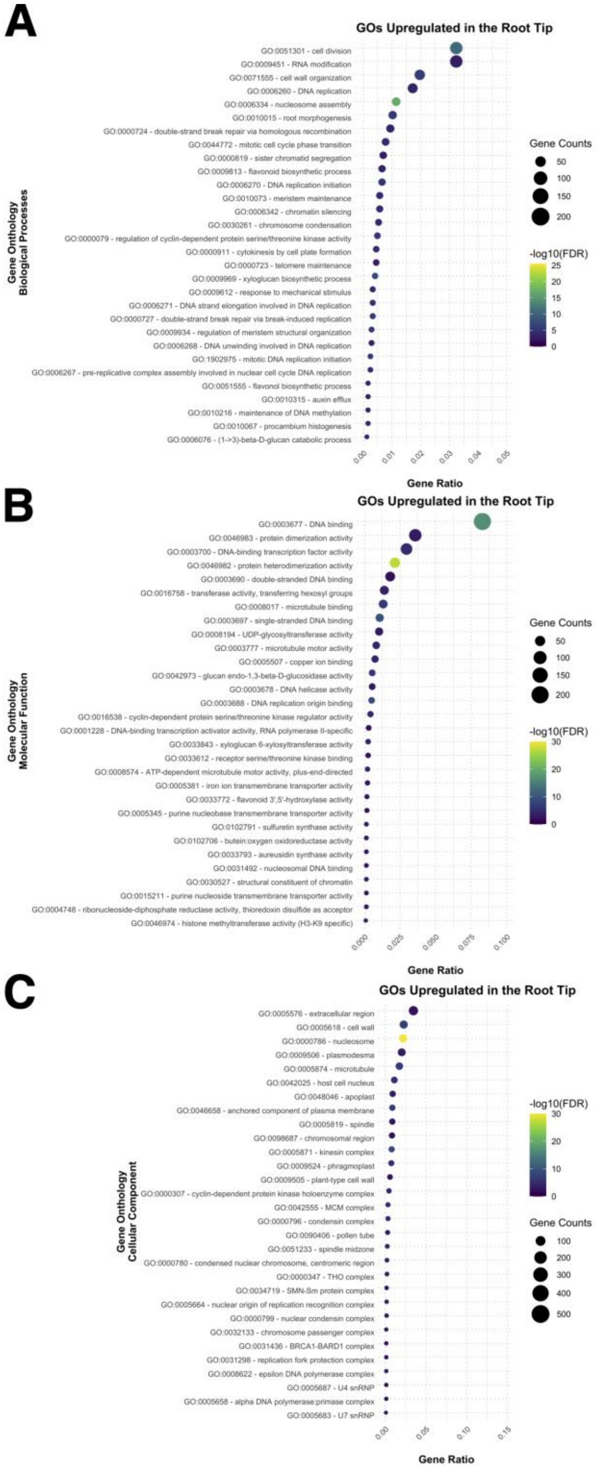

Supplementary Figure 2. Gene ontology analysis for upregulated transcripts in the root tip: biological processes (A; expanded enrichment list), molecular function (B), and cellular compartments (C).

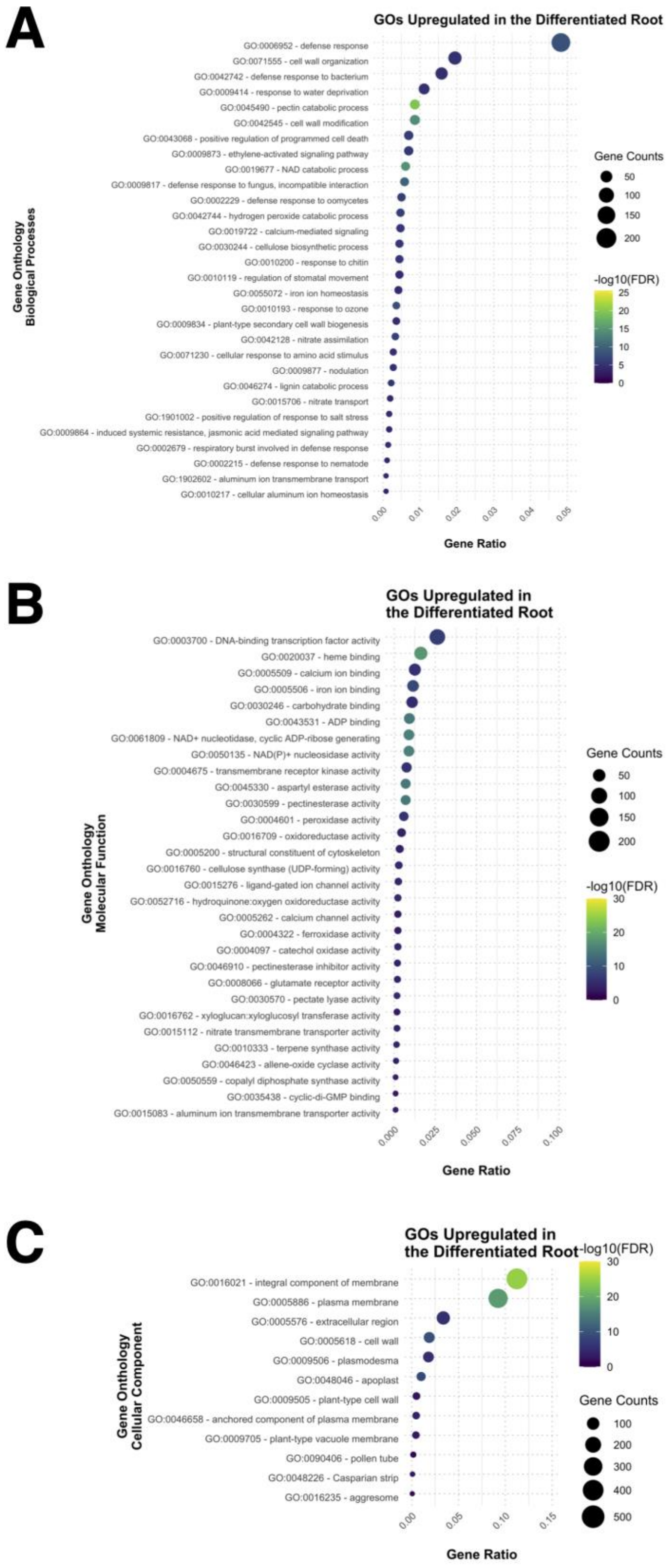

Supplementary Figure 3. Gene ontology analysis for upregulated transcripts in the differentiated root: biological processes (A; expanded enrichment list), molecular function (B), and cellular compartments (C).

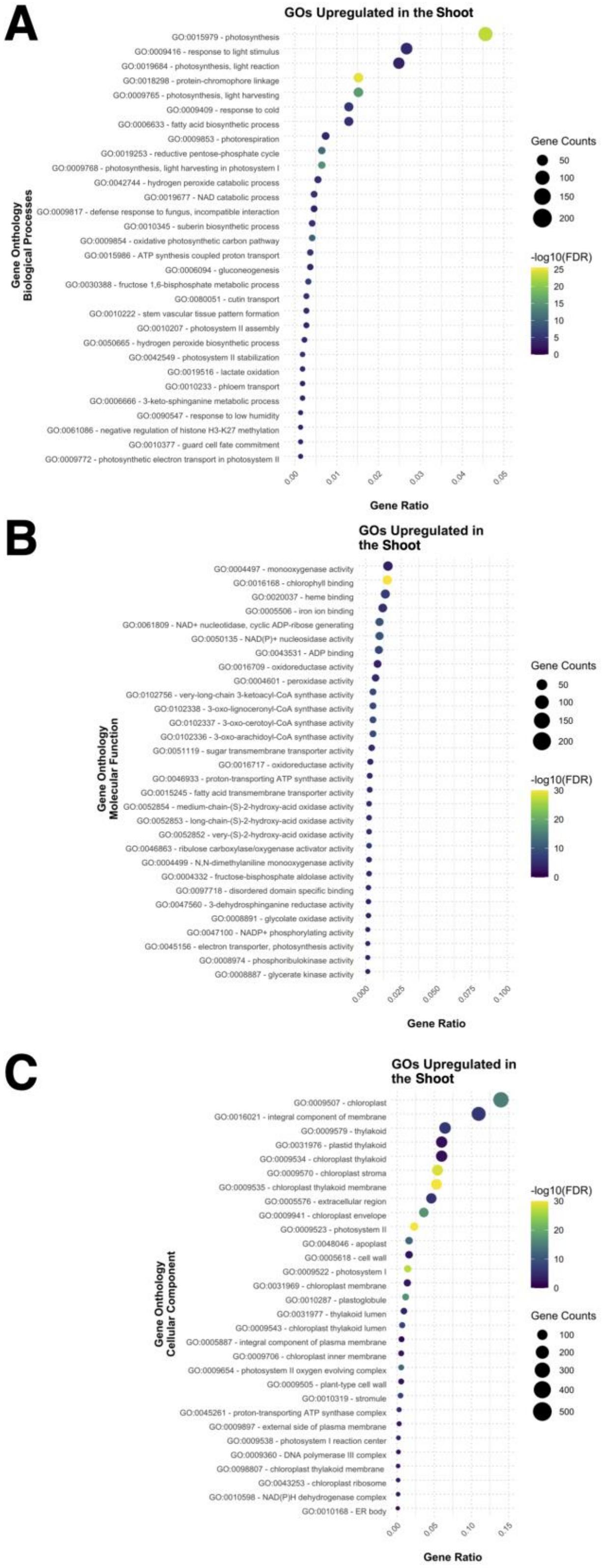

Supplementary Figure 4. Gene ontology analysis for upregulated transcripts in the leaves and shoot: biological processes (A; expanded enrichment list), molecular function (B), and cellular compartments (C).

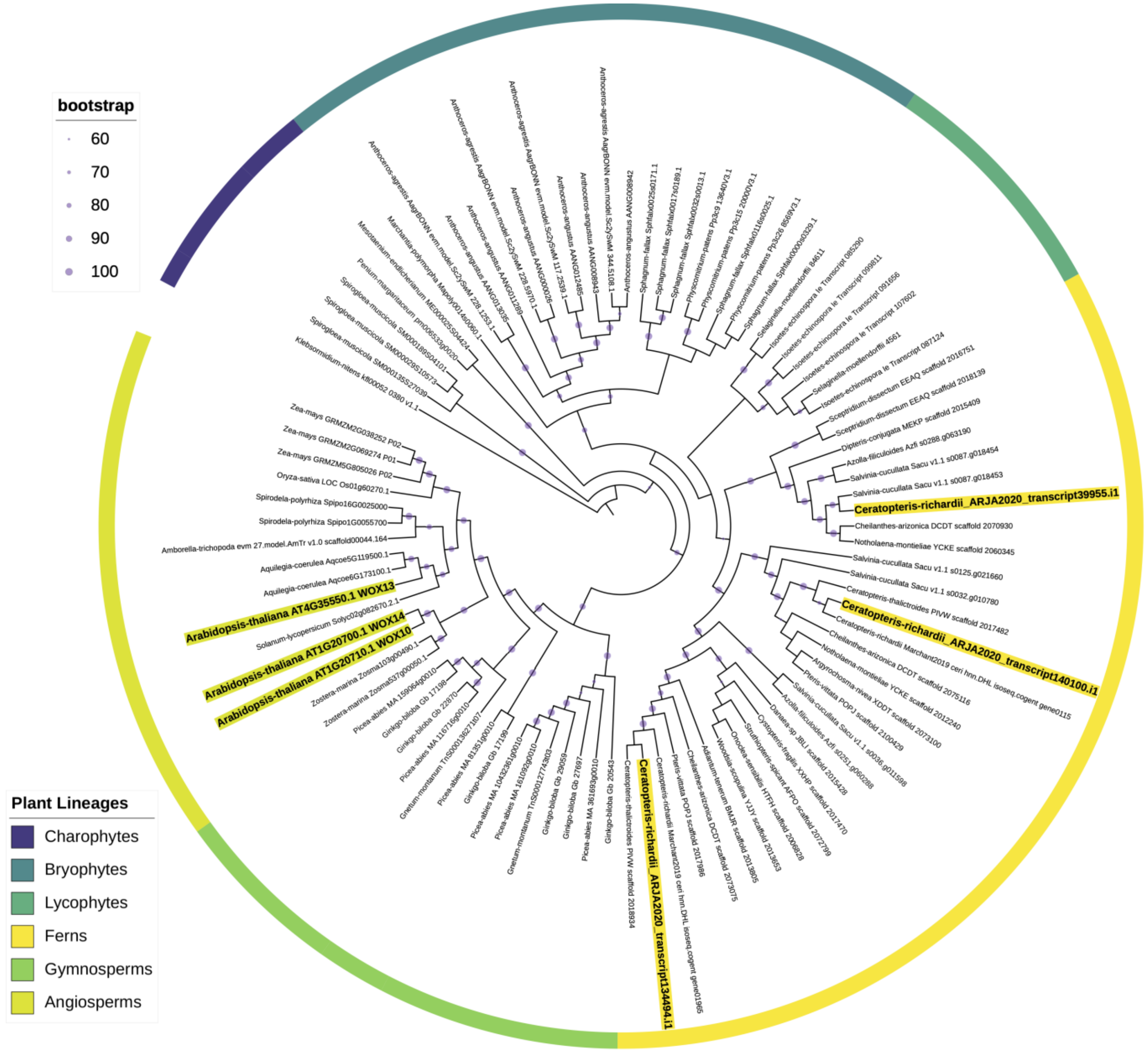

Supplementary Figure 5. Phylogenetic reconstruction for WOX13 in the Streptophyte lineage. Only bootstrap values greater than 60 are shown in the branches. Best-fit model of amino acid substitution: JTT+G. Branch lengths were not considered to improve visualization.

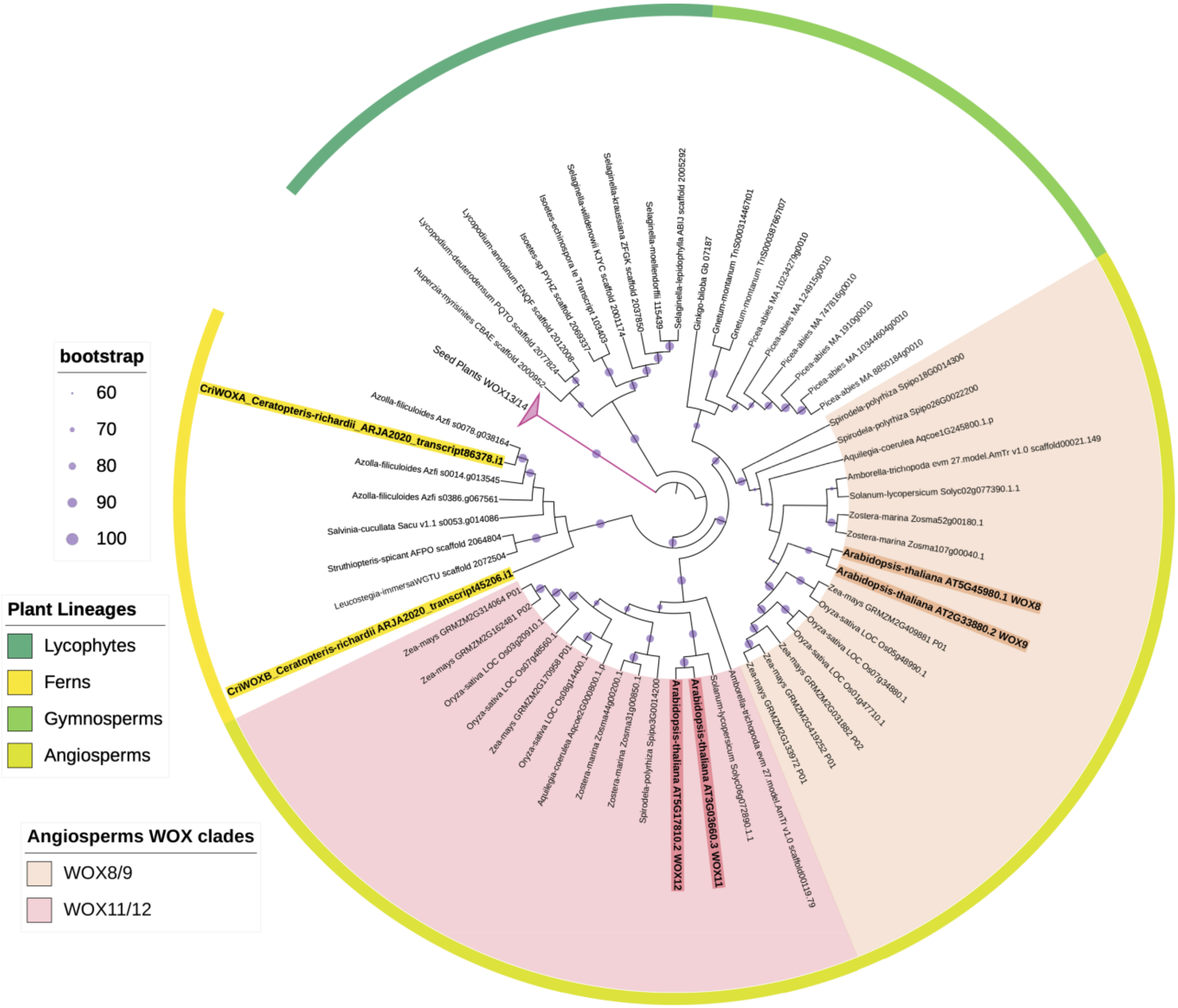

Supplementary Figure 6. Phylogenetic reconstruction for WOX9 in the Tracheophyte lineage. Only bootstrap values greater than 60 are shown in the branches. Best-fit model of amino acid substitution: JTT-DCMut+F+I+G. Branch lengths were not considered to improve visualization.

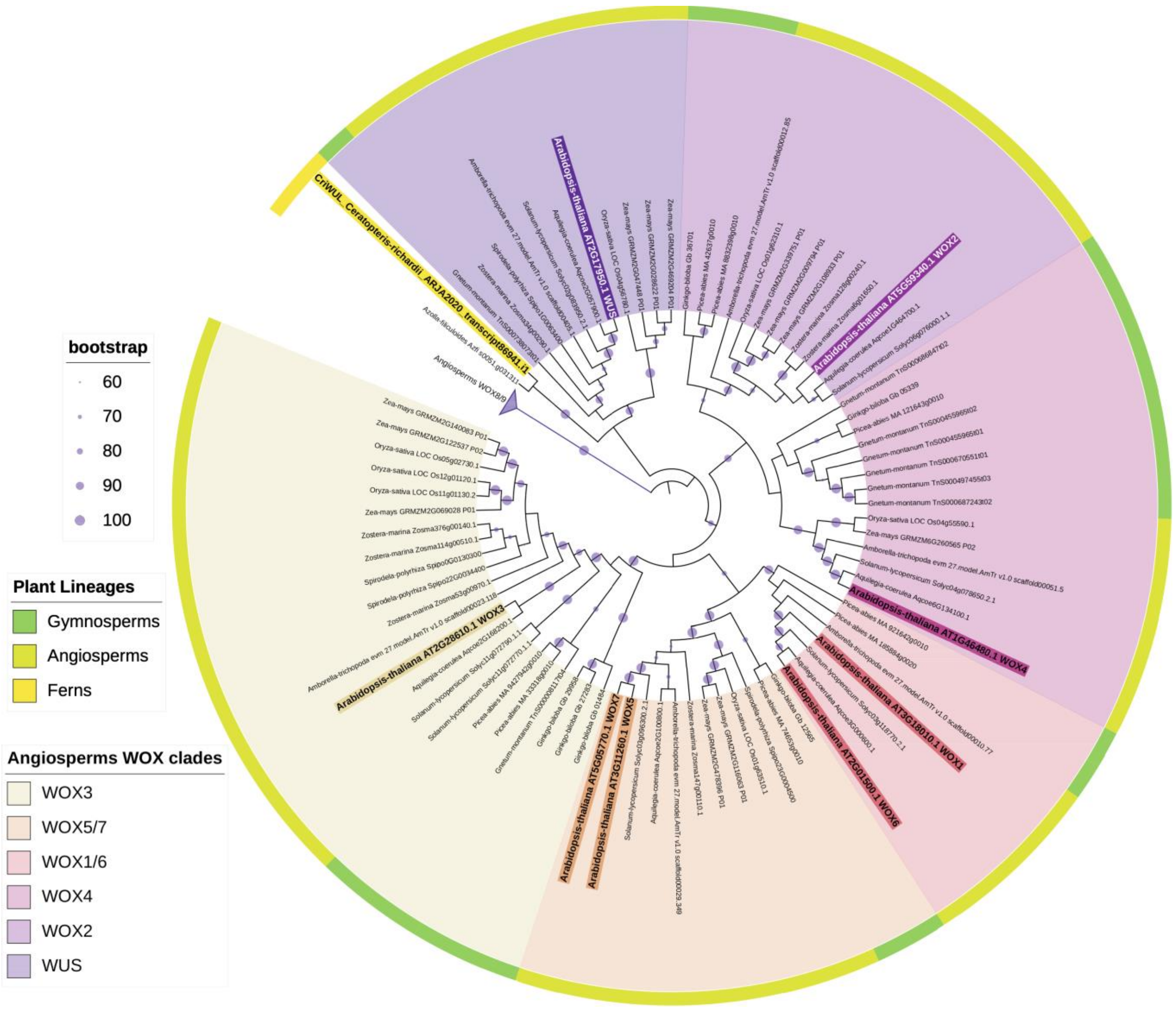

Supplementary Figure 7. Phylogenetic reconstruction for WUS in the Euphyllophyte lineage. Only bootstrap values greater than 60 are shown in the branches. Best-fit model of amino acid substitution: JTT+F+I+G. Branch lengths were not considered to improve visualization.

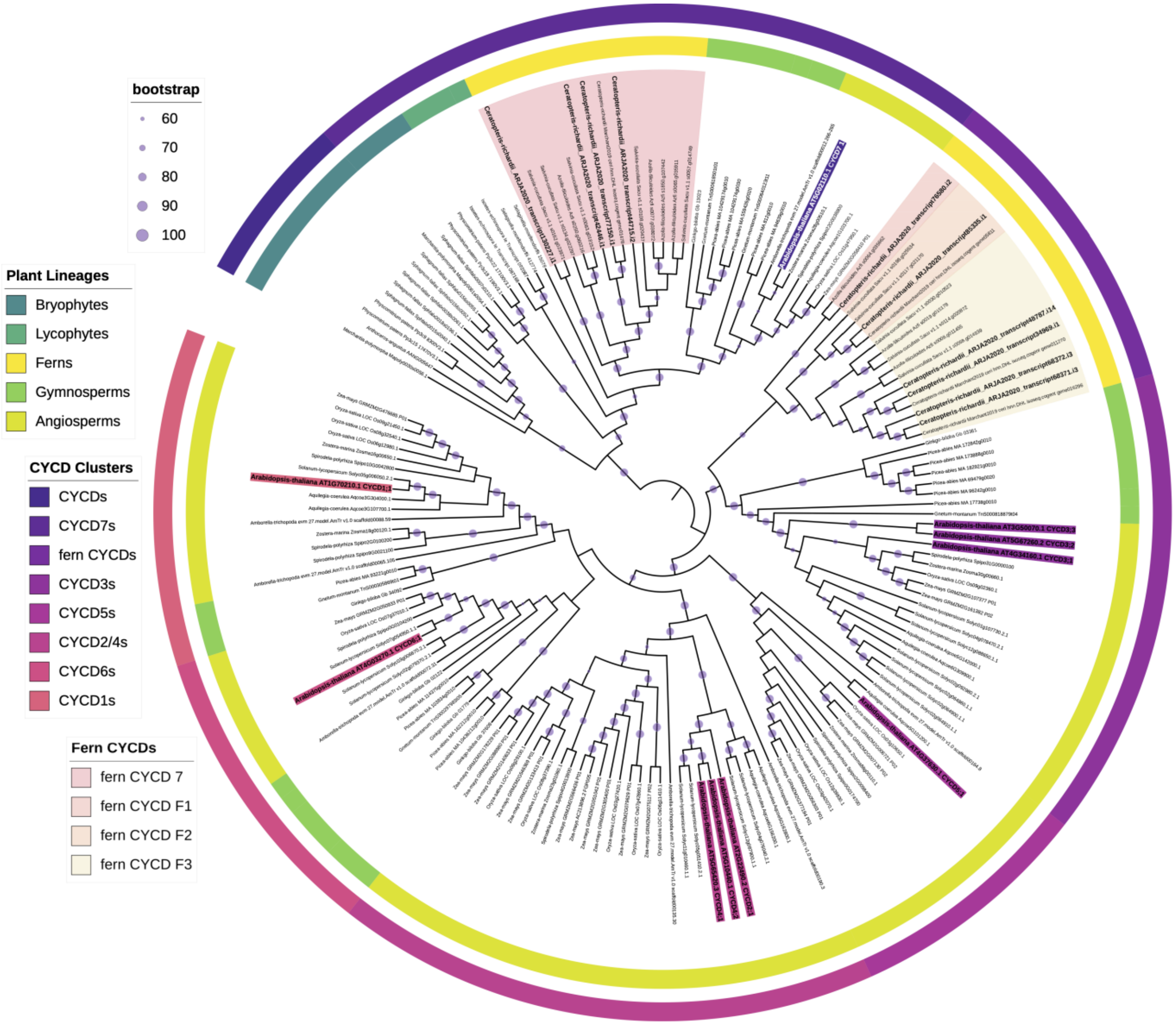

Supplementary Figure 8. Phylogenetic reconstruction for all the CYCDs in the Streptophyte lineage. Only bootstrap values greater than 60 are shown in the branches. Best-fit model of amino acid substitution: JTT+F+G. Branch lengths were not considered to improve visualization.

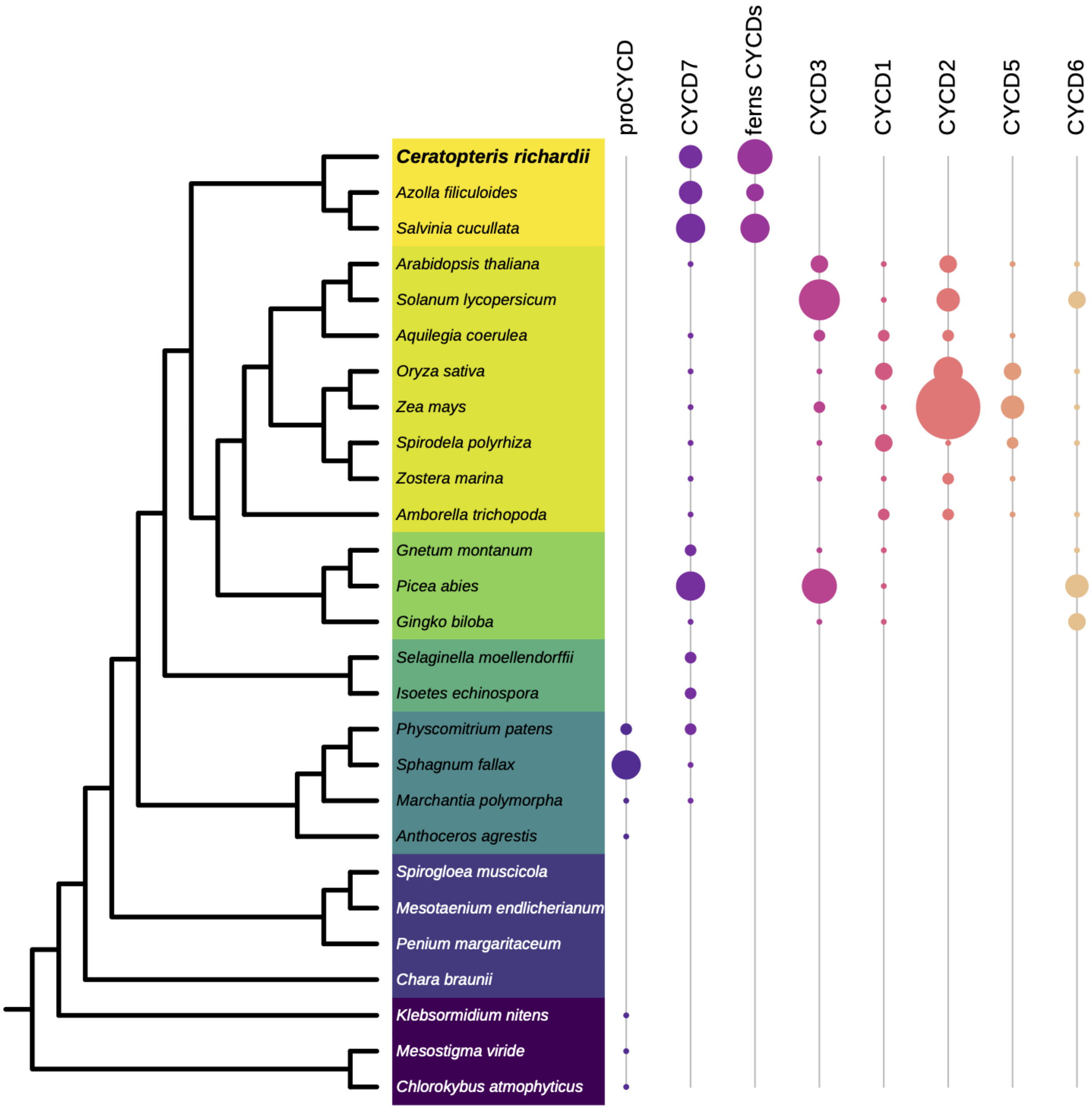

Supplementary Figure 9. Schematic representation of the presence and absence of orthologs for the different CYCDs clades throughout plant phylogeny.

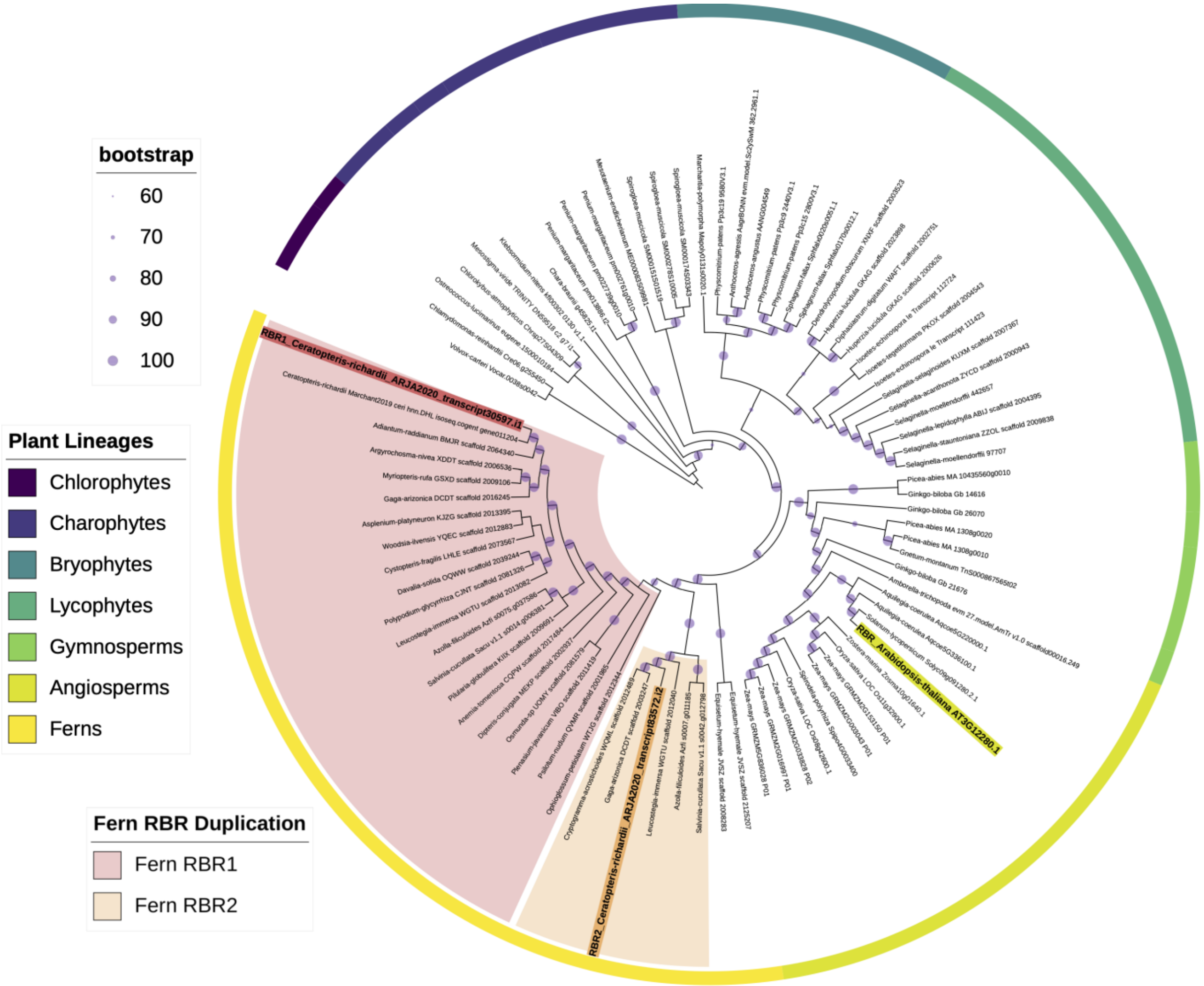

Supplementary Figure 10. Phylogenetic reconstruction for RBR in the Viridiplantae lineage. Only bootstrap values greater than 60 are shown in the branches. Best-fit model of amino acid substitution: JTT+F+I+G. Branch lengths were not considered to improve visualization.

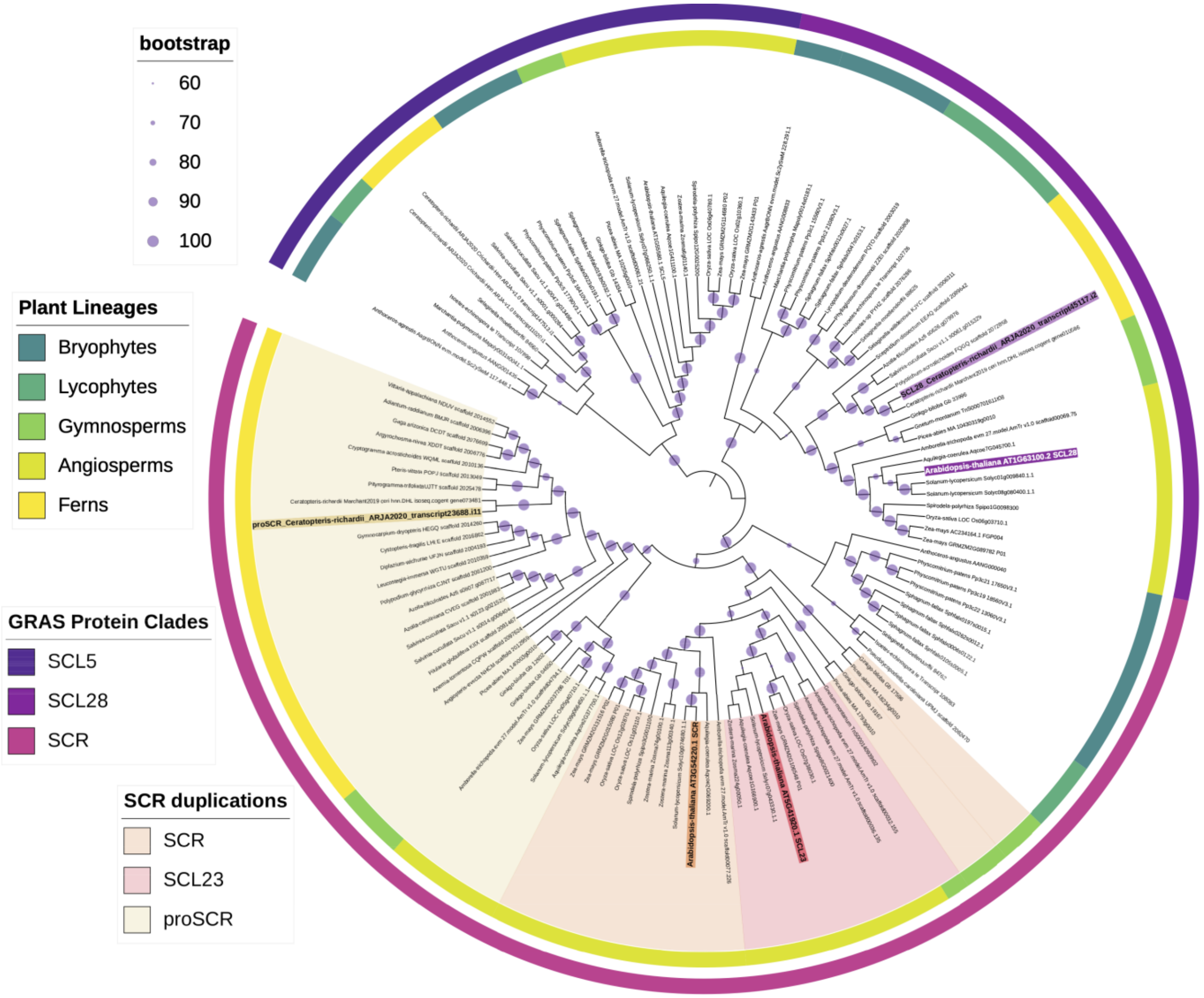

Supplementary Figure 11. Phylogenetic reconstruction for SCR in the Streptophyte lineage. Only bootstrap values greater than 60 are shown in the branches. Best-fit model of amino acid substitution: JTT+F+I+G. Branch lengths were not considered to improve visualization.

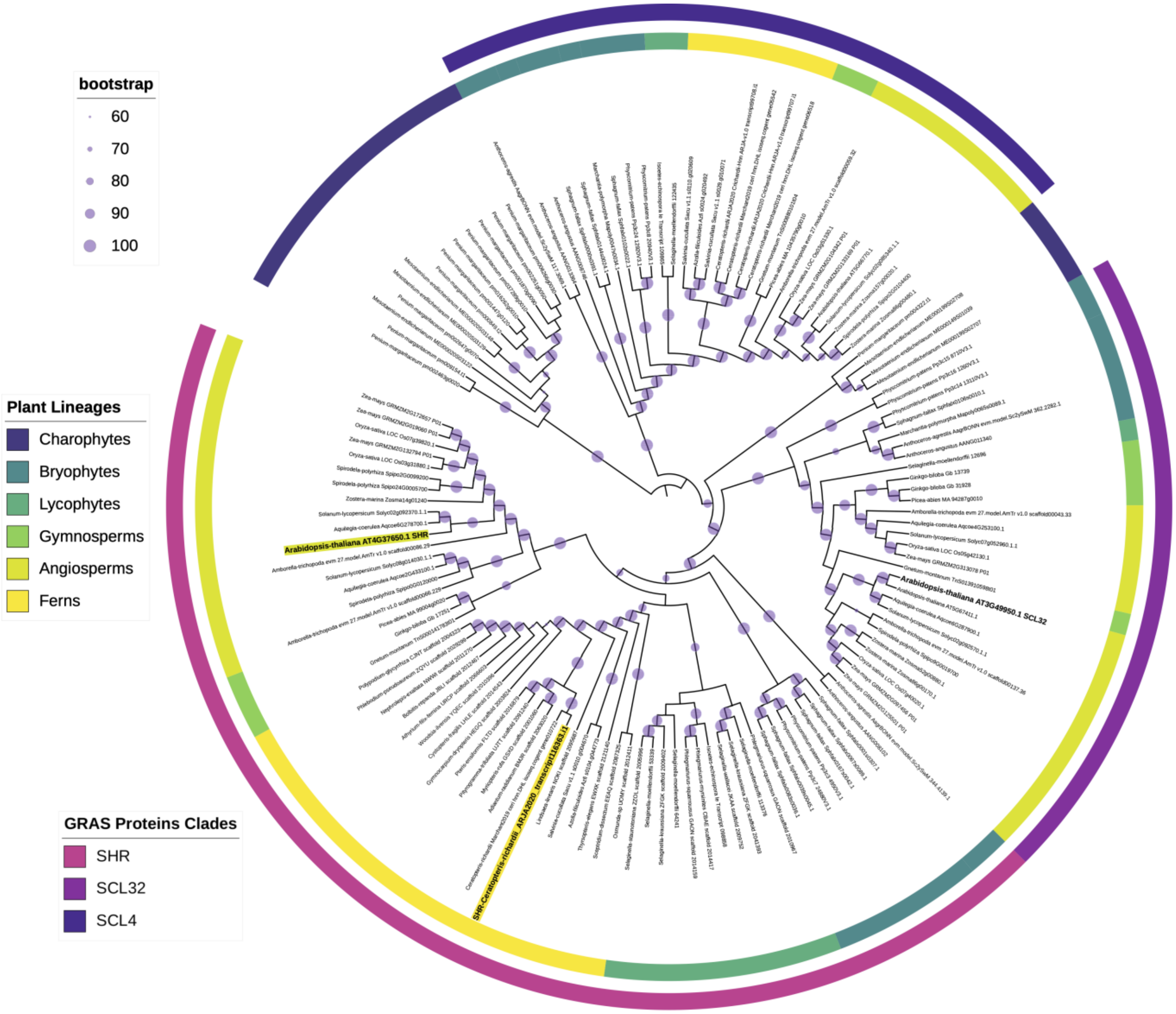

Supplementary Figure 12. Phylogenetic reconstruction for SHR in the Streptophyte lineage. Only bootstrap values greater than 60 are shown in the branches. Best-fit model of amino acid substitution: JTT+F+I+G. Branch lengths were not considered to improve visualization.

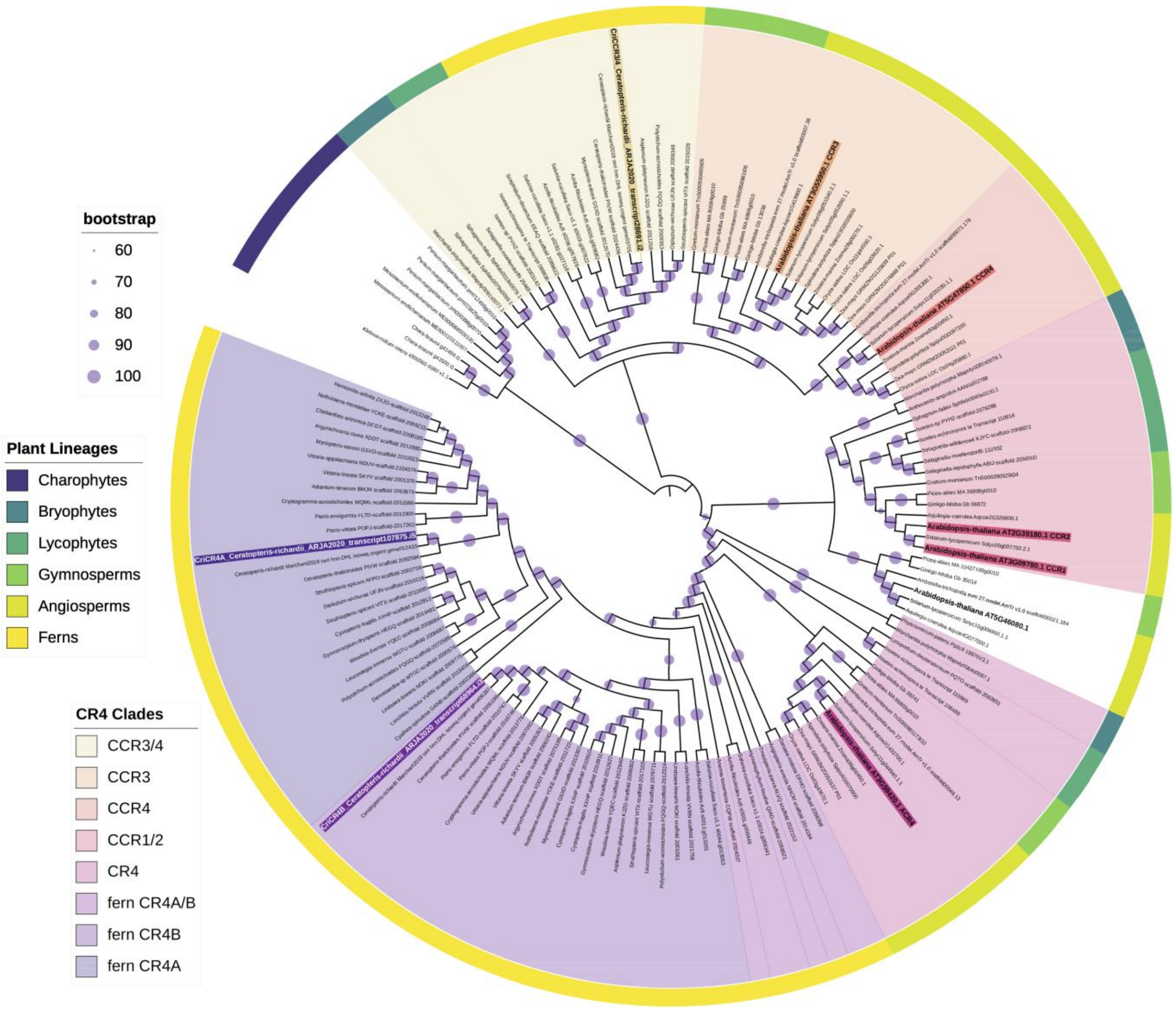

Supplementary Figure 13. Phylogenetic reconstruction for ACR4 in the Streptophyte lineage. Only bootstrap values greater than 60 are shown in the branches. Best-fit model of amino acid substitution: JTT+I+G. Branch lengths were not considered to improve visualization.

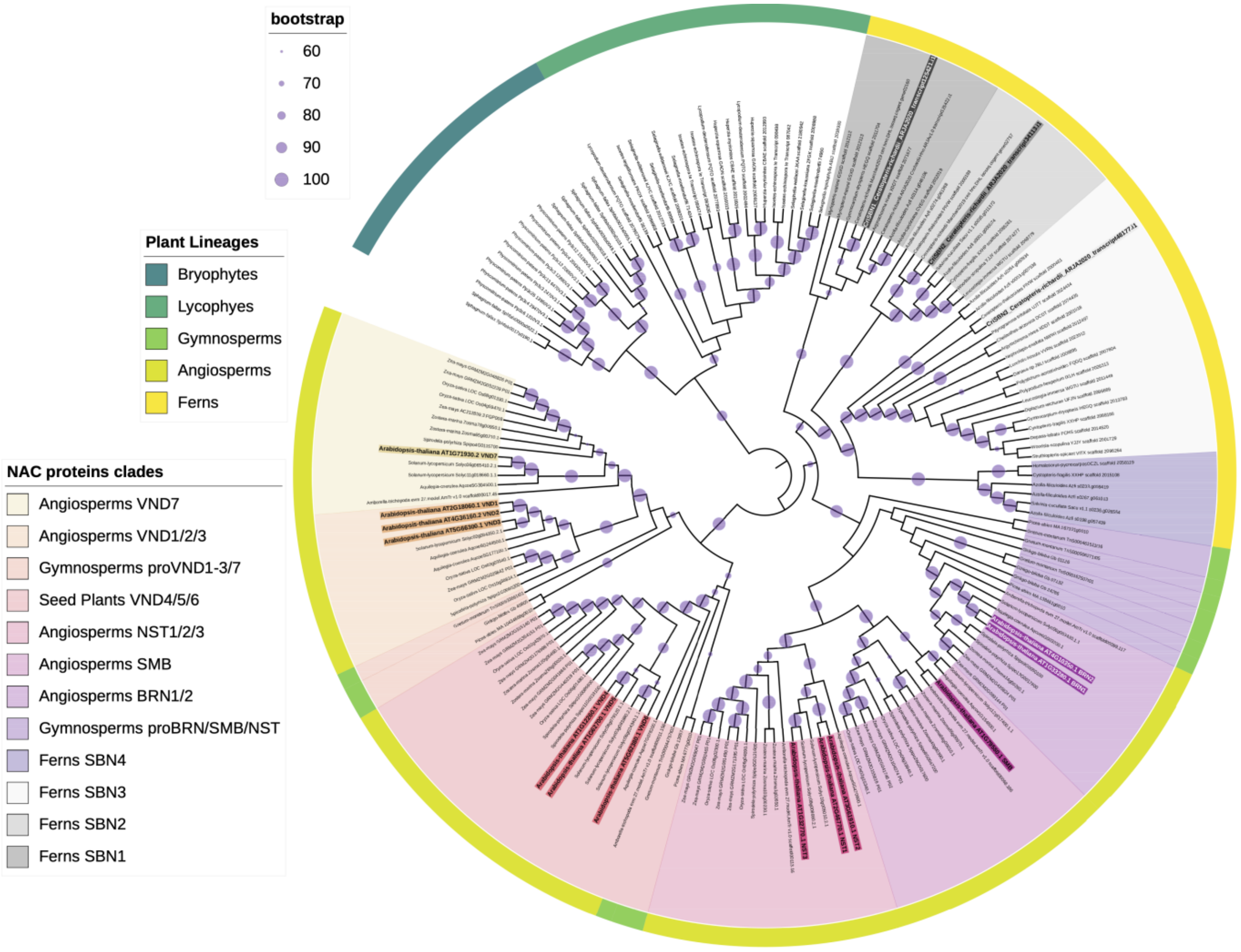

Supplementary Figure 14. Phylogenetic reconstruction for SMB in the Streptophyte lineage. Only bootstrap values greater than 60 are shown in the branches. Best-fit model of amino acid substitution: JTT+I+G. Branch lengths were not considered to improve visualization.

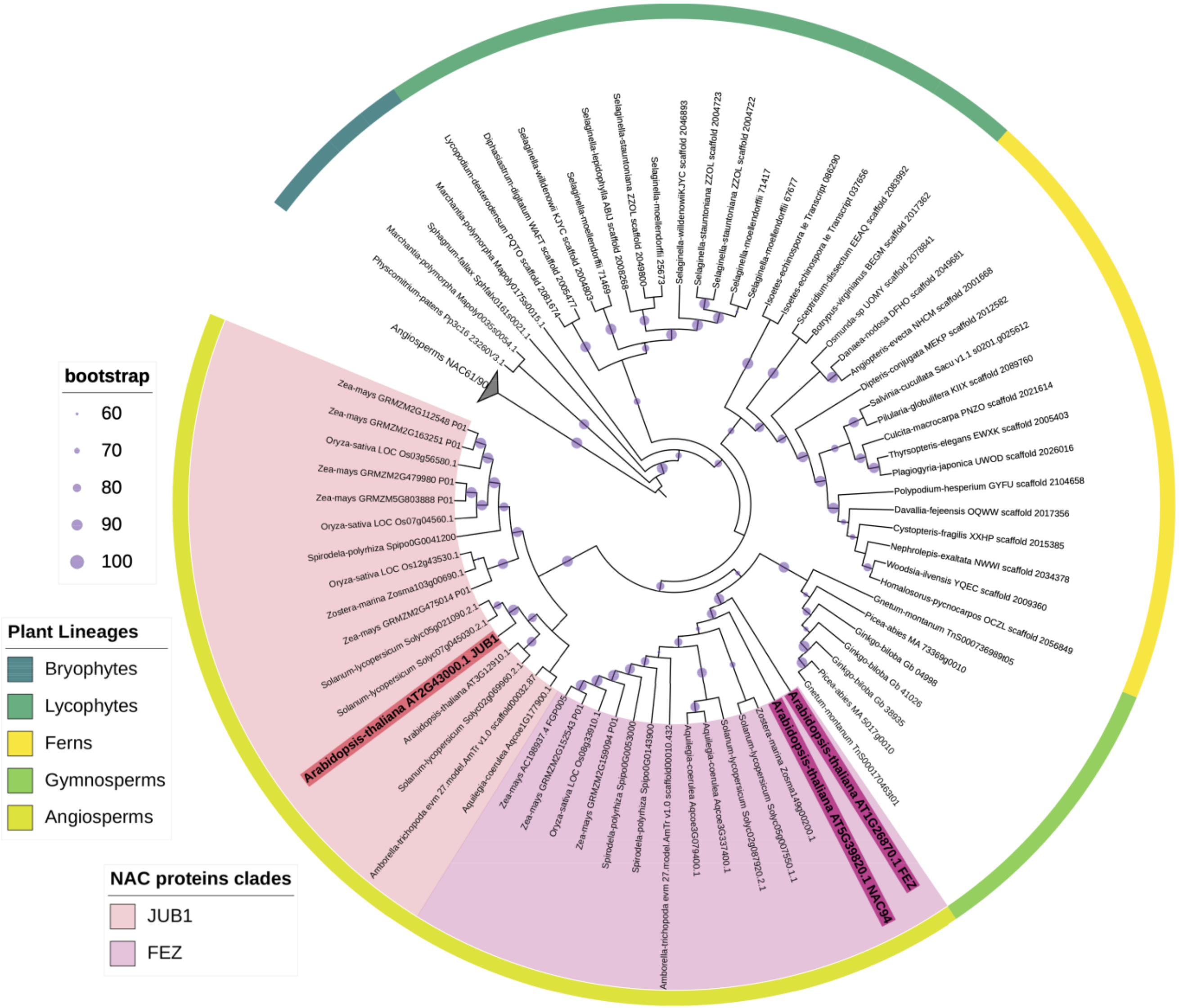

Supplementary Figure 15. Phylogenetic reconstruction for FEZ in the Streptophyte lineage. Only bootstrap values greater than 60 are shown in the branches. Best-fit model of amino acid substitution: JTT+F+I+G. Branch lengths were not considered to improve visualization.

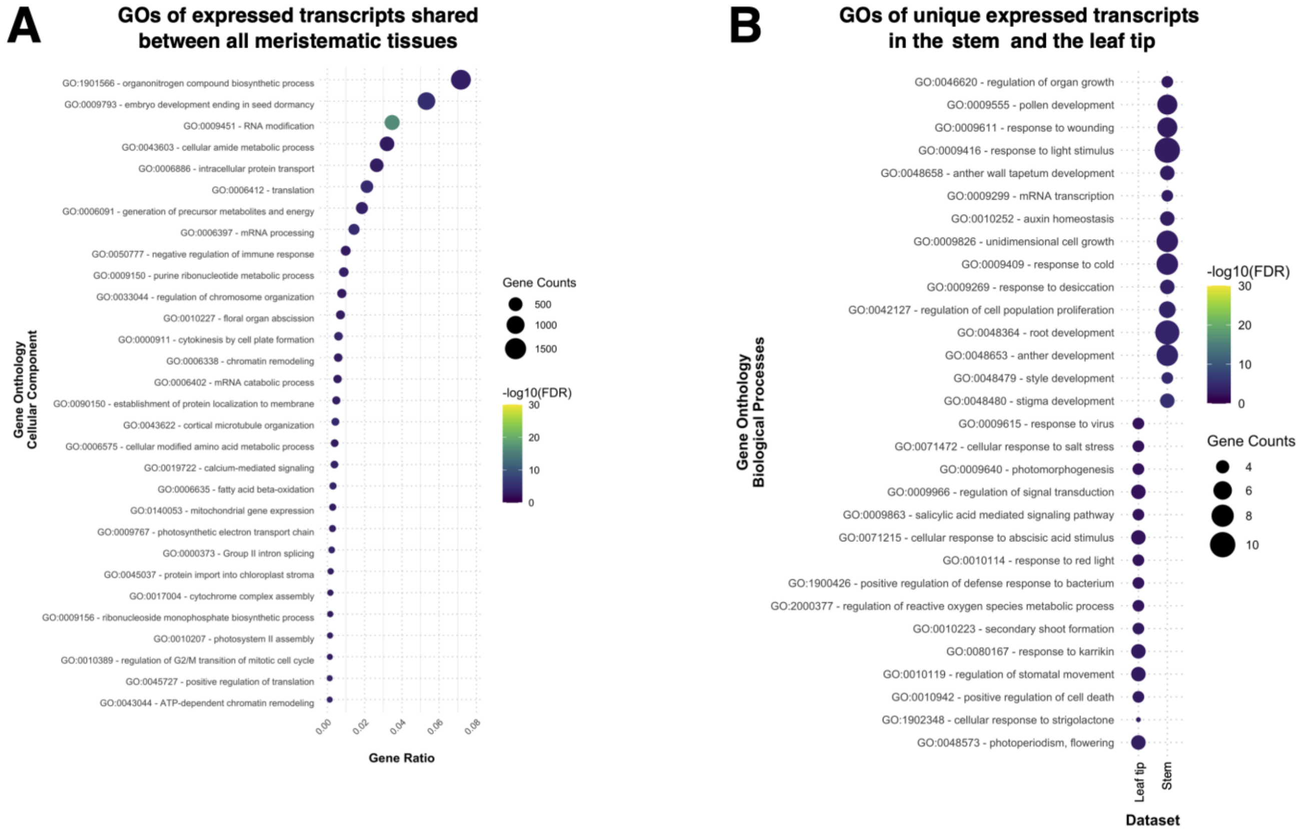

Supplementary Figure 16. Analysis from the meristematic tissues of Ceratopteris sporophytes. (**A**) Gene ontology analysis exhibiting the biological processes found shared in all meristematic tissues. (**B**) Gene ontology analysis showing the biological processes found to be enriched in other meristematic tissues. These categories were obtained from specifically expressed transcripts in the stem (including the SAM) or the leave tips.
